# PRAMEL12 orchestrates spermiogenesis to ensure male fertility in mice

**DOI:** 10.1016/j.jbc.2026.111392

**Published:** 2026-03-20

**Authors:** Nana Li, Xiao Wang, Hong Li, Zhengpin Wang

**Affiliations:** Shandong Provincial Key Laboratory of Development and Regeneration, School of Life Sciences, Shandong University, Qingdao, China

**Keywords:** PRAMEL12, spermiogenesis, chromatin remodeling, sperm morphogenesis, infertility

## Abstract

Spermiogenesis, the terminal phase of spermatogenesis, involves complex morphological and molecular changes essential for sperm maturation. While PRAMEL12, a cancer/testis antigen PRAME (preferentially expressed antigen of melanoma) family member, is implicated in spermatogenesis, its specific functions remain poorly understood. Here, global *Pramel12* deletion in mice causes complete male infertility. Mutant males exhibit severe spermiogenesis defects, including impaired spermatid nuclear condensation, spermiation failure, and loss of sperm individualization. Consequently, sperm counts are drastically reduced, motility is severely impaired, and morphological abnormalities are markedly increased. Single-cell RNA-Seq reveals dysregulation of key genes governing sperm chromatin condensation and nuclear maturation in *Pramel12*-null spermatids. Proteomic profiling shows significant alterations in proteins essential for sperm structure, function, and manchette assembly. Critically, histochemical and protein profile analyses reveal a defective histone-to-protamine transition, characterized by aberrant histone modifications (including elevated H3K4me3, H3K9me3, H3K27me3, H3K23ac, and reduced H4K8ac), impaired TNP1/TNP2/PRM2 incorporation, and increased retention of core histones (H2A, H3, and H4) in mature sperm. Collectively, our findings establish PRAMEL12 as an essential regulator of spermiogenesis. Its deficiency disrupts histone-to-protamine exchange, causing abnormal sperm morphogenesis and functional defects that impair sperm quality and lead to male infertility. This identifies a novel mechanism for defective sperm chromatin condensation and male infertility.

Long-term, steady-state spermatogenesis relies on the self-renewal and differentiation of spermatogonial stem cells into progenitor spermatogonia, which subsequently develop into mature haploid spermatozoa (1, 2). This process encompasses three distinct phases: spermatogonial mitosis, spermatocyte meiosis, and spermiogenesis (3). Following their production in the testes, spermatozoa acquire motility, undergo maturation, and gain the ability to fertilize ovulated eggs as they transit through the epididymis and female reproductive tract (4). Male fertility requires the continuous production of sufficient numbers of motile and morphologically normal sperm. Deficiencies in any of these parameters lead to male subfertility or infertility (5, 6, 7). A combination of deficits in all three metrics results in oligoasthenoteratozoospermia syndrome, the most common cause of male subfertility or infertility, characterized by reduced sperm count, impaired motility, and abnormal sperm morphology. The molecular basis of oligoasthenoteratozoospermia syndrome has not been fully elucidated.

The preferentially expressed antigen of melanoma (PRAME) protein family belongs to a class of cancer/testis antigens that are predominantly expressed in the testis and a variety of tumors, playing roles in immunity and reproduction (8, 9, 10, 11). PRAME family proteins feature leucine-rich repeat domains and LxxLL motifs, which are versatile structural elements facilitating protein–protein interactions in numerous molecular recognition processes (12, 13, 14). The mouse *Prame* gene family, one of the largest in the genome, contains approximately 90 paralogs and pseudogenes located on chromosomes 2 and 4 (15, 16). Different *Prame* gene family members exhibit distinct tissue-specific expression patterns in mice, including in the testis (*Pramex1*, *Pramel1*) (17, 18), ovary (*Oogenesin1–4*) (19, 20, 21), both testis and ovary (*Pramex2*, *Pramel3*, *Pramel12*, and *Pramel13*) (18, 22), and in embryos and primordial germ cells (*Pramel4-7*) (23, 24). While the PRAME family has been extensively studied in cancer biology, its roles in gametogenesis and reproduction remain largely underexplored. Emerging data on the spatiotemporal expression patterns and subcellular localization of PRAME family members in testicular germ cells and mature spermatozoa strongly suggest their involvement in regulating spermatogenesis and sperm maturation (18, 25, 26, 27, 28).

Few gene-edited mouse models have been employed for functional studies of PRAME family members. A prior study demonstrated that conditional deletion of *Pramex1* in male germ cells using *Stra8-Cre* mice resulted in increased germ cell apoptosis at P7 and P14, seminiferous tubule degeneration at P21 and P35, and significant spermatogonial arrest in 5% to 7% of seminiferous tubules in mature mutant mice, ultimately leading to a reduction in germ cells during spermatogenesis (29). A more recent study analyzing mouse models with either global or conditional ablation of *Pramel1* reported that PRAMEL1 regulates spermatogonial development by inhibiting retinoic acid (RA) signaling during spermatogenesis (30). In addition, double KO mice for *Pramex1* and *Pramel1* were generated, and these mutants exhibited more severely reduced fecundity, a higher proportion of Sertoli cell–only tubules, and fewer undifferentiated spermatogonia (31), suggesting a synergistic repression of the RA signaling pathway during spermatogenesis. Our previous research indicated that genetic ablation of PRAMEF12 disrupts spermatogonial maintenance, halts spermatogenesis, and leads to a Sertoli cell–only phenotype and male sterility (32). Despite these advances, systematic analysis of PRAME paralogs using gene-edited mouse models remains limited, particularly concerning their roles in spermatogenesis and sperm maturation.

This study explored the crucial role of PRAMEL12 in spermatogenesis using a genetic model with global deletion of the *Pramel12* gene in mice. *Pramel12* deficiency caused male infertility because of impaired spermiogenesis. In *Pramel12*-null mice, this impairment manifested as a decreased sperm count, severely affected motility, and increased morphological abnormalities. Spermiogenesis disruption in these mice included defective spermatid transformation, impaired spermiation, and loss of sperm individualization. *Pramel12*-deficient testes showed dysregulation of genes involved in sperm chromatin condensation and nuclear differentiation, along with that of related histone-modifying proteins. Furthermore, the loss of *Pramel12* led to an increase in sperm histones (H2A, H3, and H4) and a decrease in TNP1 and PRM2, suggesting a defective histone-to-protamine (PRM) transition. Collectively, these findings establish PRAMEL12 as an essential regulator of spermatogenesis, with its deficiency leading to defective spermiogenesis and male infertility.

## Results

### *Pramel12* is required for spermiogenesis and male fertility in mice

To investigate the role of PRAMEL12 in spermatogenesis, *Pramel12* transcript levels were initially analyzed using published single-cell RNA-Seq (scRNA-Seq) and RNA-Seq datasets from adult mouse testes (33, 34). *Sall4*, a spermatogonia-specific marker gene, was exclusively expressed in spermatogonia, confirming the reliability of the sequencing data. *Pramel12* transcripts were predominantly expressed in spermatocytes and spermatids (Fig. S1, *A* and *B*). Due to the lack of commercially available antibodies against PRAMEL12, we performed *in situ* hybridization on mouse testes at postnatal days 8 (P8), 12 (P12), and 4 months of age. This analysis confirmed *Pramel12* expression in spermatocytes and round spermatids (RSs) (Fig. S1*C*).

To explore the physiological function of PRAMEL12 in spermatogenesis and male fertility, *Pramel12*-null mice were generated using CRISPR–Cas9 technology. Two single-guide RNAs targeting intron 2, prior to the start codon in exon 3, and two additional single-guide RNAs targeting exon 5, after the stop codon, were used. This process resulted in a founder line lacking the entire coding sequence, confirmed by Sanger sequencing (Fig. S2*A*). *Pramel12*-null mice were subsequently bred to homozygosity and validated *via* genomic PCR (Fig. S2*B*). No *Pramel12* transcript was detected in the testes of *Pramel12*-null mice by quantitative RT–PCR (qRT–PCR) or *in situ* hybridization (Fig. S2, *C* and *D*), indicating successful generation of the null model.

**Figure 1:**
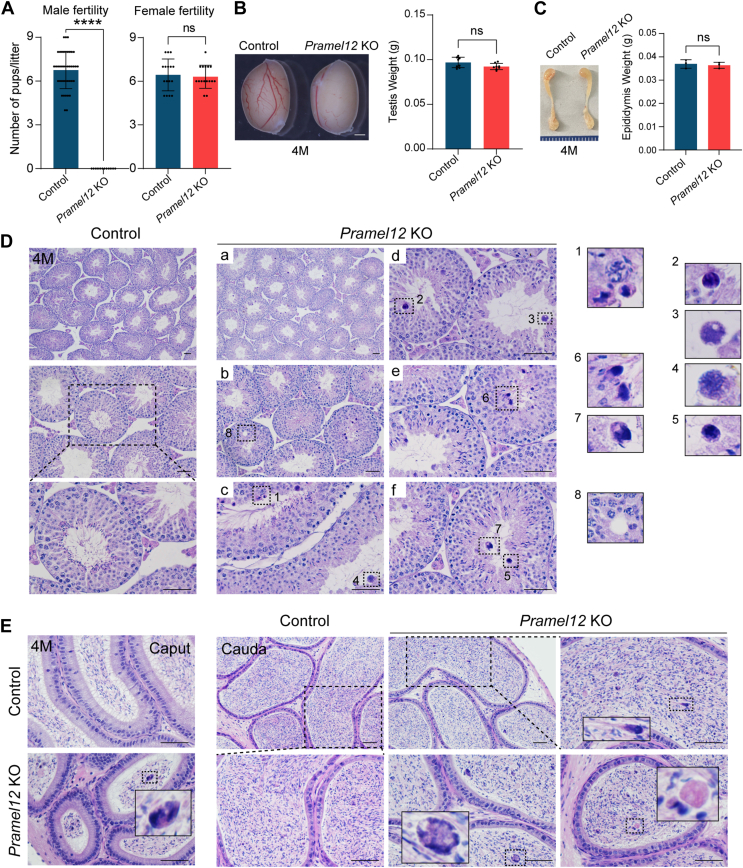
**PRAMEL12 is required for spermiogenesis and male fertility.***A*, fertility tests in adult *Pramel12* KO male (*left panel*) and female (*right panel*) mice. Mean number of pups ± SD; ns, no significance; ∗∗∗∗*p* < 0.0001. *B*, morphology (*left panel*) and weight (*right panel*) of testes from 4-month-old control and *Pramel12* KO mice. The scale bar represents 1 mm. Data are presented as mean ± SD; ns, no significance. *C*, same as (*B*), but for epididymides. (*D*) H&E-stained sections of control and *Pramel12* KO testes from 4-month-old mice. Higher-magnification images corresponding to the boxed regions. The scale bar represents 50 μm. *E*, H&E-stained cross-sections of caput and cauda epididymis regions in 4-month-old control and *Pramel12*-null mice. Higher-magnification images corresponding to the boxed regions. The scale bar represents 50 μm. Representative of n = 3 (*D*, *E*) independent biological replicates with similar results per condition. PRAME, preferentially expressed antigen of melanoma.

Fertility tests revealed that adult *Pramel12*-null homozygous males were completely infertile and produced no offspring, whereas mutant females showed normal fecundity (Fig. 1*A*). This indicates that PRAMEL12 is essential for male fertility. To determine the underlying cause of male infertility, testes and epididymides from adult (4-month-old) males underwent morphological and histological analyses. Both testes and epididymides displayed normal size, with no significant weight differences observed between *Pramel12*-null males and control littermates (Fig. 1, *B* and *C*). Histological analysis of *Pramel12*-null testes revealed intact seminiferous tubules and normal spermatocytogenesis, as evidenced by the presence of spermatogonia, spermatocytes, and spermatids. However, spermiogenesis was defective (Figs. 1*D* and S3*A*), characterized by frequent aggregates of elongating spermatids (Fig. 1*D*, #1), large residual bodies from immature spermatozoa within the lumen (Fig. 1*D*, #2–5 and Fig. S3*A*), the persistence of cytoplasmic remnants adhering to immature elongating spermatids (Fig. 1*D*, #6–7), and small vacuoles (Fig. 1*D*, #8). Both caput and cauda epididymides contained sloughed seminiferous tubule contents, including round germ cells (DDX4 positive/SYCP3 [synaptonemal complex protein 3] negative; Fig. S3*B*) and cytoplasmic remnants consistent with residual bodies (Fig. 1*E*). The cauda epididymides of *Pramel12*-null males additionally exhibited aggregated immature spermatozoa (spermatozoa bundles) (Fig. 1*E*). Collectively, these defects demonstrate that PRAMEL12 disruption impairs spermiogenesis, leading to male infertility.

To determine whether the spermiogenesis defects in young (4-month) *Pramel12*-null males progressed with age, we analyzed older (12-month) mice. Strikingly, *Pramel12*-null testes and epididymides were significantly smaller and lighter than those of control littermates (Fig. S4, *A*–*D*). Histological analysis confirmed this deterioration. In contrast to the relatively intact tubules seen at 4 months, 12-month-old *Pramel12*-null testes displayed severe anomalies, including a significant reduction in tubular diameter, a disorganized epithelial structure, and a high frequency of vacuoles across all stages of the seminiferous cycle (Fig. S4, *E*–*I*). Consequently, the epididymides of these older mutants contained predominantly sloughed germ cells and very few spermatozoa (Fig. S4*J*). Immunostaining confirmed that the sloughed germ cells were RSs, as evidenced by DDX4-positive/SYCP3-negative staining in the epididymis (Fig. S5). Collectively, these findings demonstrate the crucial role of PRAMEL12 in spermatogenesis and highlight that PRAMEL12 ablation leads to a significant reduction in spermatogenic cells and defective spermiogenesis.

To determine the impact of PRAMEL12 on spermatogenic cells in older mutants, we measured the abundance of undifferentiated (PLZF^+^) and differentiated (KIT^+^) spermatogonia. Immunofluorescence and statistical analysis revealed that these populations were comparable in *Pramel12*-null and control testes (Fig. S6, *A* and *B*), indicating intact spermatogonial development. Analysis of meiotic markers SYCP3 and γH2AX showed significantly fewer spermatocytes in *Pramel12*-deficient testes (Fig. S6, *C* and *D*), demonstrating severely impaired meiotic development. Furthermore, germ cell loss in mutant testes, indicated by vacuolization, was confirmed by TUNEL assays demonstrating significantly more apoptotic cells per tubule in 12-month-old mutants (Fig. S7, *A* and *B*). TUNEL and γH2AX costaining further revealed apoptosis in multiple germ cell populations, including general germ cells, elongated spermatozoa (Fig. S7*C*), and a subset of meiotic spermatocytes that were positive for both TUNEL and γH2AX (Fig. S6*E*). To analyze meiosis, we prepared chromosome spreads from spermatocytes of 12-month-old control and *Pramel12*-null testes and stained them for SYCP3 and γH2AX. Although *Pramel12*-null spermatocytes progressed from leptotene to diplotene, quantitative analysis revealed an accumulation in pachytene and a reduction in diplotene stages compared with controls (Fig. S6, *F* and *G*), indicating impaired meiotic progression. These results indicate that *Pramel12* deficiency results in increased apoptosis, drastic spermatocyte loss, and impaired meiotic progression.

### *Pramel12* deficiency reduces sperm count, impairs sperm motility, and increases the incidence of sperm morphological abnormalities

**Figure 2:**
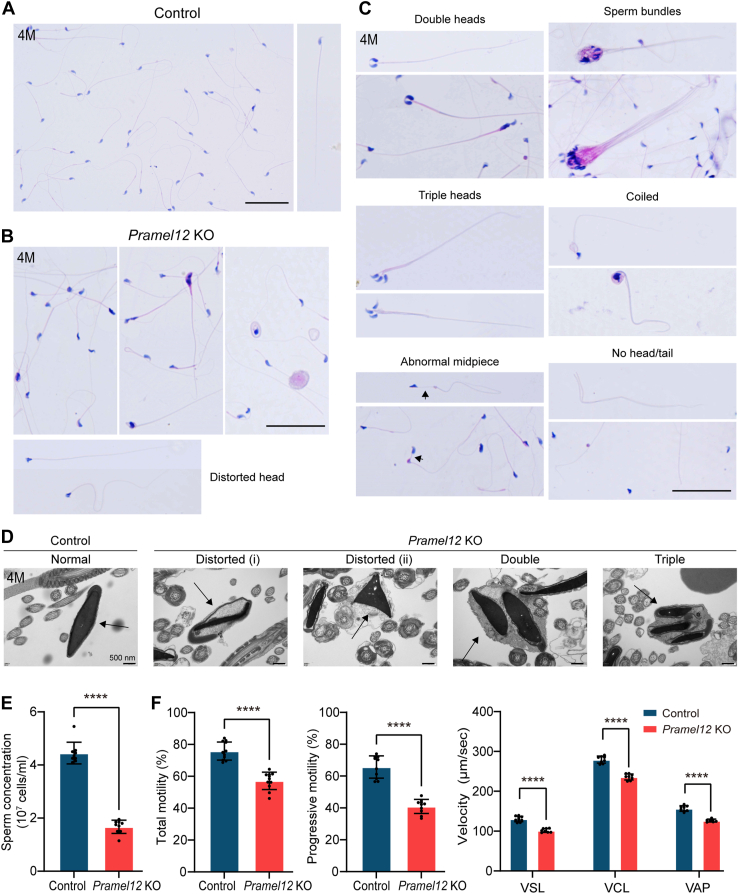
**Sperm morphology and parameter assessment of *Pramel12*-null mice.***A*–*C*, H&E staining of epididymal sperm from 4-month-old control and *Pramel12* KO mice. The scale bar represents 50 μm. *D*, TEM analysis of epididymal sperm from adult control and *Pramel12*-null male mice. The scale bar represents 500 nm. *Arrows* indicate sperm nuclei. *E*, sperm concentration of cauda epididymides from adult control and *Pramel12*-deficient mice. *F*, computer-assisted sperm analysis of sperm motility, progressive motility, straight-line velocity (VSL), curvilinear velocity (VCL), and average path velocity (VAP) from adult control and *Pramel12*-null mice. Data are presented as mean ± SD; ∗∗∗∗*p* < 0.0001. Representative of n = 3 (*A*–*C*) independent biological replicates with similar results per condition. PRAME, preferentially expressed antigen of melanoma; TEM, transmission electron microscopy.

Given that PRAMEL12 deletion leads to infertility in early adult (4-month-old) males with only moderate effects on spermatocyte and spermatogenic cell development, we focused on this age group to identify the underlying causes. To assess whether infertility was due to sperm defects, we analyzed sperm from the cauda epididymis of 4-month-old *Pramel12*-null mice. Morphologically abnormal sperms were predominantly observed in *Pramel12*-null males, with only rare occurrences in control littermates. *Pramel12*-null males exhibited a high frequency of sperm abnormalities, including distorted heads, double/triple heads, malformed midpieces, coiled necks, aggregated spermatozoa bundles, and decapitated sperm (Figs. 2, *A*–*C* and S8*A*). Quantitative analysis revealed that over 90% of spermatozoa displayed morphological defects, primarily involving malformed heads or structural abnormalities, such as midpiece defects (Fig. S8*B*). To examine these abnormalities in detail, transmission electron microscopy (TEM) was performed. Compared with the normal sperm heads of control mice, *Pramel12*-null males displayed abnormal head morphology, including distorted, duplicated, and even triplicated heads (Fig. 2*D*). In addition, multinucleated sperm heads, frequently accompanied by cytoplasmic remnants, were observed in these mutants (Fig. 2*D*). Sperm counts in the epididymides of *Pramel12*-null males were significantly lower than in controls (Fig. 2*E*). Using computer-assisted sperm analysis, sperm parameters were evaluated. Results showed that sperm motility and progressive motility were significantly reduced in *Pramel12*-null males (Fig. 2*F*). Additional parameters, including straight-line velocity, curvilinear velocity, and average path velocity, were also dramatically decreased in *Pramel12*-null males (Fig. 2*F*). These results indicate that PRAMEL12 is crucial for the formation of morphologically normal sperm and that its deficiency results in reduced sperm count and impaired sperm motility.

The high incidence of sperm head abnormalities, suggesting acrosomal defects, prompted us to assess acrosomal integrity in cauda epididymal sperm using FITC-conjugated peanut agglutinin (PNA) staining. While control mice showed well-formed acrosomes on sperm heads, most *Pramel12*-null sperm had abnormally shaped acrosomes, with detectable PNA binding (Fig. S9, *A* and *D*). The abnormal acrosome morphology in *Pramel12*-deficient sperm likely results from defective sperm head morphogenesis. Because acrosome exocytosis is required for fertilization, its frequency was quantified by calculating the proportion of PNA-negative (acrosome-reacted) sperm relative to Hoechst-positive (total viable) sperm. Fluorescent staining and statistical analysis demonstrated that spontaneous acrosome reaction rates were significantly higher in both noncapacitated and capacitated sperm from *Pramel12*-null mice compared with controls (Fig. S9, *B*, *C* and *E*). These results indicate that *Pramel12* deficiency leads to abnormal acrosome morphology and elevated acrosome reaction rates in male mice.

Sperm defects (malformed morphology, reduced count, and premature acrosomal reaction) evident in 4-month-old mutant males persisted and worsened by 12 months of age, resulting in significantly lower sperm counts and a higher incidence of acrosome reactions (Fig. S10, *A*–*D*).

### Disorganized mitochondrial sheath, disrupted flagellar axonemes, and reduced fertilization capacity of *Pramel12*-deficient sperm

**Figure 3:**
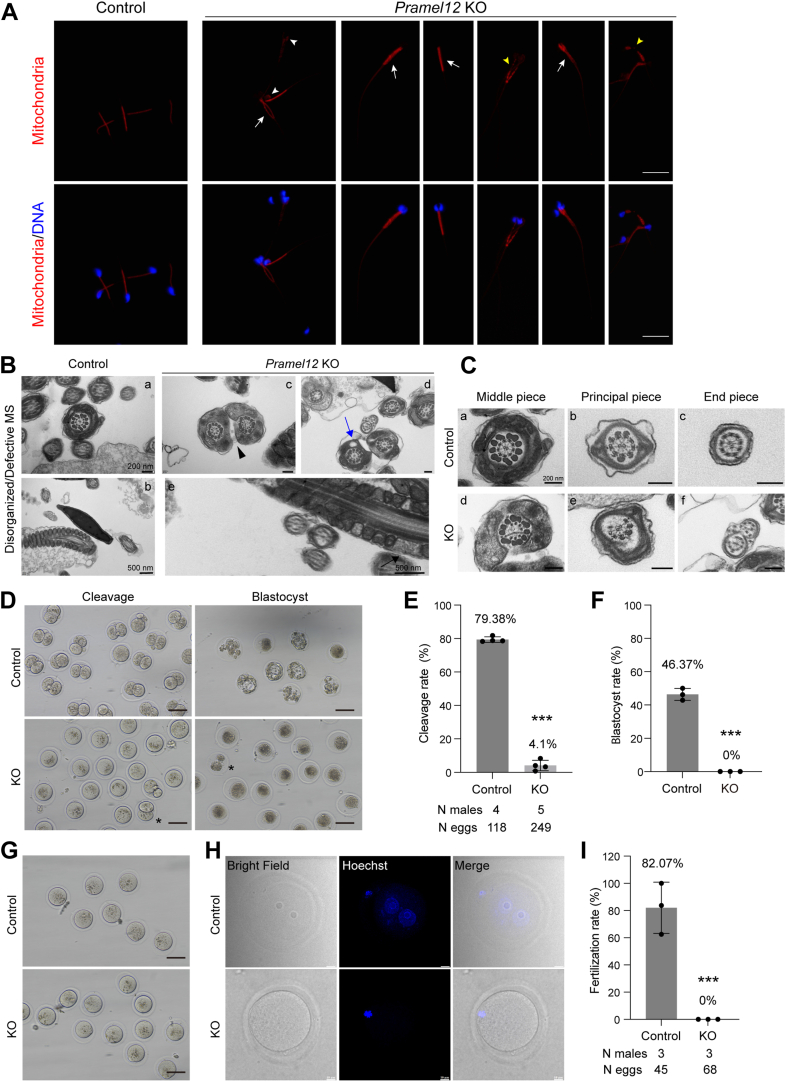
***Pramel12* KO mice exhibit an abnormal mitochondria sheath, a disrupted flagellar axoneme, and reduced fertilization capacity.***A*, representative images of mitochondria staining in cauda epididymal sperm from 4-month-old control and *Pramel12* KO mice. DNA was stained with Hoechst 33342. *White arrowheads* indicate mitochondrial signals on the sperm heads. *White arrows* indicate abnormal mitochondrial signals. *Yellow arrowheads* indicate discontinuous mitochondrial signals. The scale bar represents 50 μm. *B*, TEM images of epididymal sperm showing cross-sectional and longitudinal views of the midpiece. *Arrow*, *arrowhead*, and *blue arrow* indicate abnormal mitochondria. Image (*D*) is an enlarged view of the region (ii) in Figure 2*D*. The scale bars represent 200 nm and 500 nm. *C*, TEM images showing defective axoneme structure in *Pramel12*-deficient sperm. Image (*A*) is an enlarged view of region (*A*) in Figure 3*B*. Image (*B*) is an enlarged view of region (*B*) in Figure 5*C*. Image (*D*) is an enlarged view of region (*C*) in Figure 3*B*. Image (*F*) is an enlarged view of region (*D*) in Figure 3*B*. The scale bar represents 200 nm. *D*, representative images of embryos after IVF with epididymal sperm from adult control and *Pramel12* KO mice, cultured for 1 day (*left panels*) and 3 days (*right panels*). *Asterisks* indicate two-cell embryos. The scale bar represents 100 μm. *E* and *F*, quantitative analysis of the cleavage rate (*E*) and blastocyst formation rate (*F*) following IVF with control or *Pramel12*-null sperm. Data are presented as mean ± SD; ∗∗∗*p* < 0.001. *G* and *H*, representative images of embryos collected from the ampullae of wildtype females the day after mating with control or *Pramel12*-null males (*G*) and the corresponding Hoechst 33342 staining of these embryos (*H*). The scale bars represent 100 μm in (*G*); 10 μm in (*H*). *I*, quantification of fertilization rates from the embryos shown in (*G*). Data are presented as mean ± SD; ∗∗∗*p* < 0.001. IVF, *in vitro* fertilization; PRAME, preferentially expressed antigen of melanoma; TEM, transmission electron microscopy.

Based on the multiple morphological defects and severely compromised motility observed in 4-month-old *Pramel12*-deficient sperm, we therefore investigated the functional role of PRAMEL12 in mitochondrial integrity, flagellar axoneme structure, and *in vitro* fertilization (IVF) capacity. MitoTracker staining confirmed normal midpiece-localized mitochondria in control sperm, whereas *Pramel12*-deficient sperm exhibited disrupted mitochondrial distribution (Fig. 3*A*). MitoTracker signals in the sperm head (*white arrowheads*) were indicative of defective cytoplasmic droplet removal. Furthermore, *Pramel12*-null sperm exhibited discontinuous (*yellow arrowheads*), disorganized mitochondrial patterns, which manifested as branches or aggregates (*white arrows*). TEM analysis revealed well-aligned mitochondrial sheaths in control sperm, whereas *Pramel12*-null sperm exhibited disrupted sheaths and ultrastructural anomalies, including flagella with multiple axonemes enveloped by malformed (*arrowhead*) or vacuolated (*arrows*) mitochondria (Fig. 3*B*). TEM analysis further confirmed that control sperm flagella displayed the typical "9 + 2" axonemal structure in the midpiece, principal piece, and end piece, characterized by nine peripheral microtubule doublets surrounding two central singlets. In contrast, *Pramel12*-null sperm exhibited aberrant axonemes with partial or complete loss of central singlets and/or peripheral doublets (Fig. 3*C*). To assess fertilization capacity, an IVF assay was performed. *Pramel12*-null sperm exhibited a significant reduction in fertilization ability. After 1 day of culture, approximately 4.1% of oocytes fertilized with mutant sperm had developed into two-cell embryos, compared with 79.38% in the control group (Fig. 3, *D* and *E*). Following an additional 2 days in culture, none of the two-cell embryos from the null group developed to the blastocyst stage, whereas 46.37% did so in the control group (Fig. 3*F*). Furthermore, we assessed the *in vivo* fertilization capacity of *Pramel12*-null males. After mating with wildtype females, embryos were collected from the ampulla the following day for analysis. In the control group, 82.07% of oocytes were fertilized, as indicated by the presence of both male and female pronuclei. In contrast, none of the oocytes from the null group were fertilized and remained arrested at the metaphase I stage (Fig. 3, *G*–*I*). Collectively, these results demonstrate that *Pramel12*-deficient sperm displays disorganized mitochondrial sheaths, disrupted flagellar axoneme structures, and, ultimately, a significantly reduced fertilization capacity.

### Sperm head malformations emerge during the condensing phase of spermiogenesis in *Pramel12*-deficient mice

**Figure 4:**
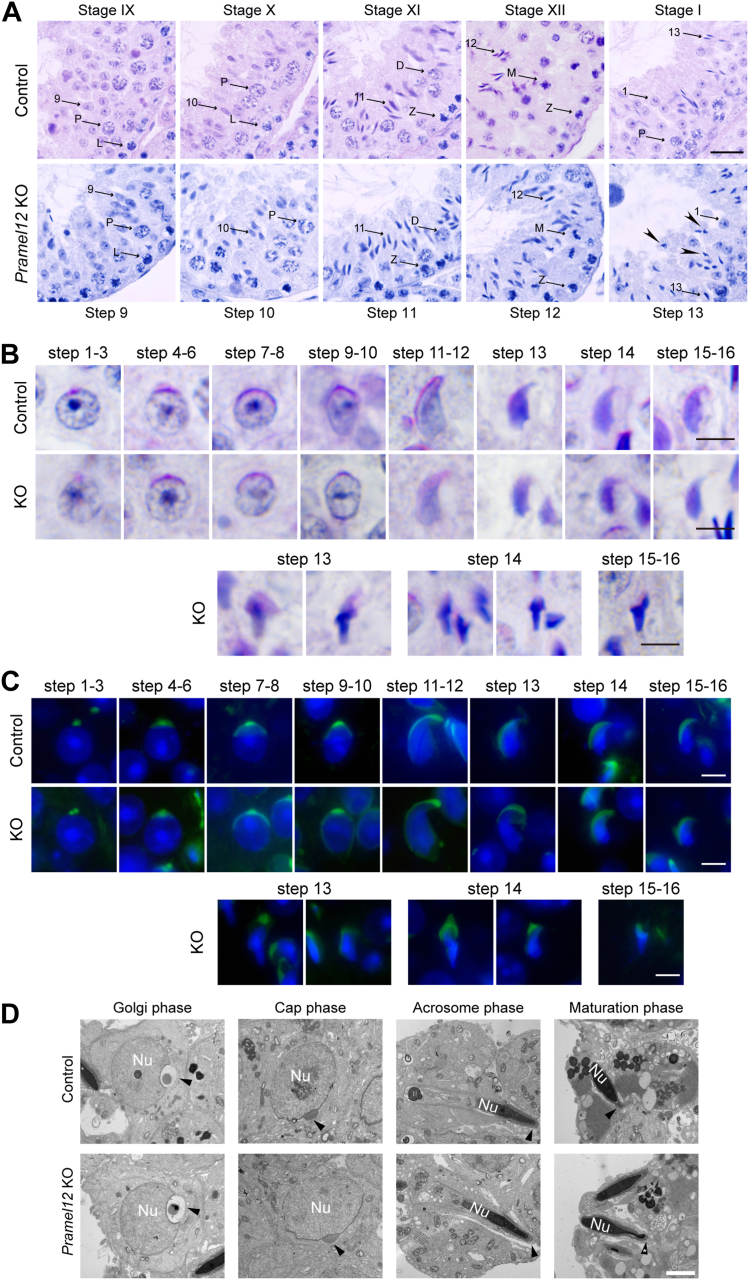
**Spermiogenic defects become detectable at the nuclear condensation phase in *Pramel12*-null mice.***A*, testicular sections from 4-month-old control and *Pramel12*-null mice were stained with HE. Tubules at different stages of the seminiferous cycle were indicated. *Arrowheads* mark elongating spermatids exhibiting abnormal nuclear morphology at condensation stage 13. The numbers indicate spermatids at the corresponding developmental stages. L, leptotene; Z, zygotene; P, pachytene; D, diplotene; and M, metaphase. The scale bar represents 50 μm. *B*, periodic acid-Schiff staining of haploid spermatids during spermiogenesis from steps 1 to 16 in 4-month-old control and *Pramel12*-null mice. Malformed step 13 spermatids were observed during nuclear condensation in *Pramel12*-null mice. The scale bar represents 5 μm. *C*, spermatids were stained with PNA and Hoechst 33342 during spermiogenesis in 4-month-old control and *Pramel12*-null mice. Malformed sperm heads and acrosomes were observed at step 13 in *Pramel12*-null mice. The scale bar represents 5 μm. *D*, representative TEM images of acrosome biogenesis at Golgi, cap, acrosome, and maturation stages in control and *Pramel12*-deficient spermatids. *Arrowheads* indicate acrosomes. The scale bar represents 2 μm. Representative of n = 3 (*A*–*C*) independent biological replicates with similar results per condition. PNA, peanut agglutinin; TEM, transmission electron microscopy.

Spermiogenesis involves the morphological remodeling of RSs into elongated spermatids through defined developmental stages (steps 1–16) (35, 36). In mice, the seminiferous epithelial cycle is divided into 12 stages, which are grouped as stages I–III, IV–VI, VII–VIII, IX–X, and XI–XII (37). To determine when spermatid morphogenetic defects arise, we tracked differentiation throughout spermiogenesis using histological and acrosomal stainings. Histological examination of stages IX–XII and I seminiferous tubules showed that spermatid head malformations in *Pramel12*-deficient testes became detectable around spermiogenesis step 13, coinciding with nuclear condensation (Fig. 4*A*). To confirm this observation, periodic acid-Schiff staining was performed on spermatids during steps 1 to 16 of spermiogenesis, a method that specifically labels acrosomal structures. Periodic acid-Schiff staining showed that sperm head malformations appeared at the condensing phase and persisted through step 16 (Fig. 4*B*), indicating sustained structural defects during late spermiogenesis. PNA staining confirmed that abnormal acrosome morphology emerged alongside head malformations from step 13 onward, corresponding to the acrosome maturation phase (Fig. 4*C*). TEM analysis revealed normal sperm head and acrosome formation through the Golgi, cap, and acrosome phases in *Pramel12*-deficient males until the maturation phase, when spermatids exhibited severe defects in both structures (Fig. 4*D*). Together, these results show that sperm head malformations arise in the condensing phase of spermiogenesis.

### Impaired spermiation and loss of sperm individualization in *Pramel12*-null mice

**Figure 5:**
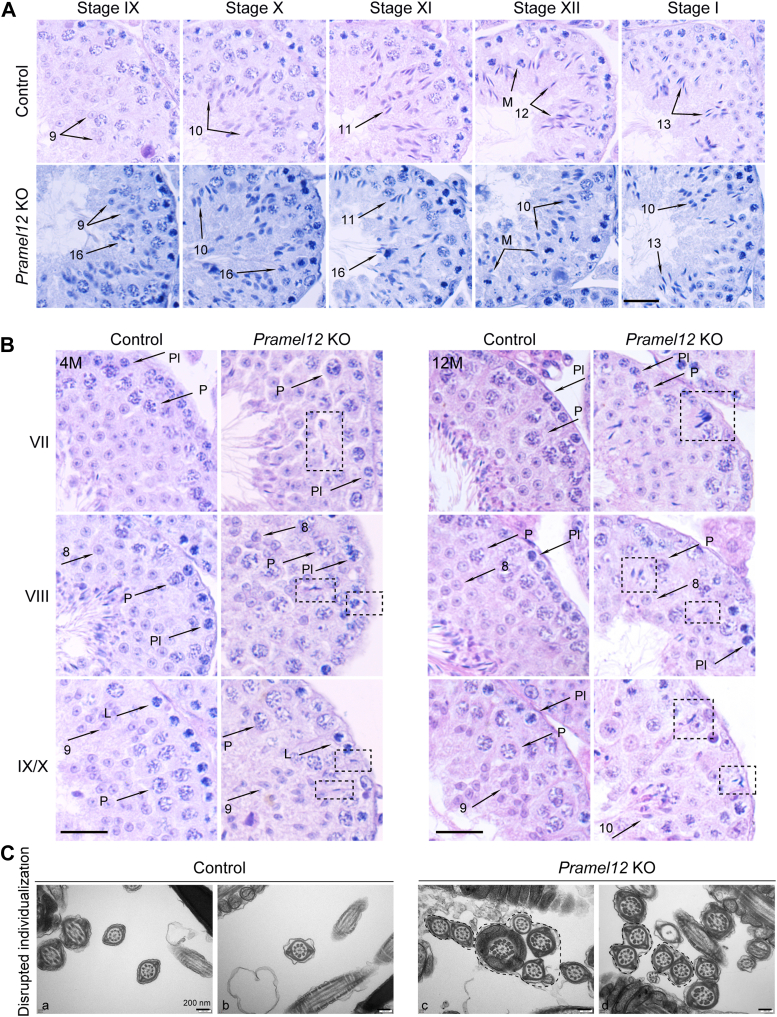
**Defective spermiation and sperm individualization in *Pramel12*-null mice.***A*, testicular sections of 4-month-old control and *Pramel12*-null mice were stained with HE. Tubules at different stages of the seminiferous cycle and spermatozoa at different developmental steps are indicated. M, metaphase. The scale bar represents 50 μm. *B*, seminiferous tubule cross-sections at stages VII, VIII, and IX/X from control and *Pramel12*-null mice at 4 months and 12 months. *Dashed rectangles* indicate elongated spermatids embedded deep in the seminiferous epithelium. The numbers indicate spermatids at the corresponding developmental stages. Pl, preleptotene; P, pachytene. The scale bar represents 50 μm. *C*, TEM images of epididymal sperm showed disrupted individualization in *Pramel12*-null mice. *Dashed lines* indicate that multiple sperm axonemes were wrapped in one cell membrane. The scale bar represents 200 nm. Representative of n = 3 (*A*, *B*) independent biological replicates with similar results per condition. TEM, transmission electron microscopy.

To further investigate the multifaceted defects in spermiogenesis, seminiferous epithelium cycle progression, spermiation efficiency, and sperm individualization were analyzed in 4-month-old *Pramel12*-null mice. During spermiation, elongated spermatid heads align along the luminal edge of the epithelium before their release from stages VII and VIII seminiferous tubules (38, 39). Histological examinations revealed that step 16 spermatids persisted in stages IX–XI tubules of *Pramel12*-null mice, whereas they were absent in controls (Fig. 5*A*). In addition, step 10 spermatids were aberrantly retained in stage XII tubules, and mixing of step 10 and 13 spermatids was observed in stage I tubules of mutant males (Fig. 5*A*), indicating disrupted seminiferous epithelial cycle progression and impaired spermiation. Histology of stages VII–X further revealed a spermiation defect in both 4- and 12-month-old *Pramel12*-null testes. Unlike controls where spermatids aligned luminally (VII and VIII) and were released (IX and X), mutant spermatids were mislocalized deep in the epithelium at VII and VIII and retained basally at IX/X, contrasting with full release in controls (Fig. 5*B*). These observations demonstrate impaired spermiation efficiency in *Pramel12*-null mice. To examine defects in sperm individualization, epididymal spermatozoa were analyzed by TEM. In controls, properly individualized spermatozoa exhibited intact plasma membrane encapsulation with a single axoneme (Fig. 5*C*). Strikingly, *Pramel12*-null spermatozoa frequently displayed multiple axonemes enclosed within a shared cytoplasmic compartment (Fig. 5*C*), indicating impaired intercellular bridge resolution during late spermiogenesis and disrupted sperm individualization. These results indicate that *Pramel12* deficiency leads to multiple defects during spermiogenesis.

### scRNA-Seq analysis of spermatid population abundance in *Pramel12*-null testes

To investigate the molecular consequences of *Pramel12* deletion during spermiogenesis, scRNA-Seq analysis was performed on 4-month-old testes using the 10x Genomics platform, comparing the transcriptomes of control and *Pramel12*-null mice. A total of 13,905 control and 11,940 *Pramel12*-deficient testicular cells passed quality control and were retained for subsequent analysis. Uniform Manifold Approximation and Projection (UMAP) and marker gene analyses were employed to identify cell types within the combined control and *Pramel12*-null testicular cells. Based on the expression patterns of known marker genes in the mouse testis, nine distinct cell types were identified. These included four germ cell populations: spermatogonia, spermatocytes, spermatids, and elongating spermatozoa; and five somatic cell populations: endothelial cells, macrophages, Leydig cells, stroma, and Sertoli cells (Fig. S11, *A* and *B*). The distribution of cell types was similar between control and *Pramel12*-null samples (Fig. S11*C*).

**Figure 6:**
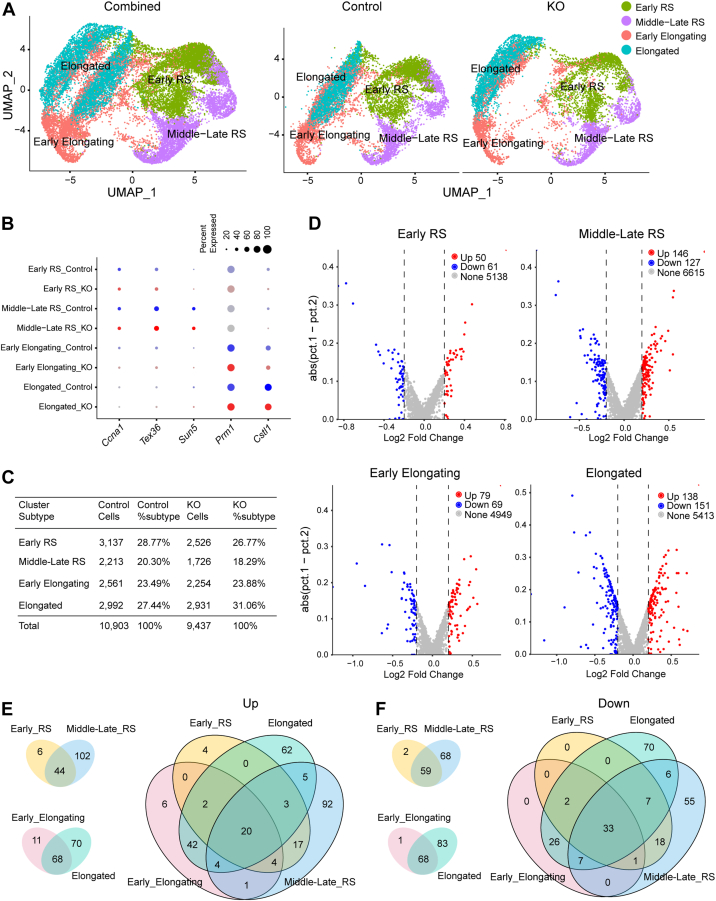
**scRNA-Seq analysis of *Pramel12*-null spermatids.***A*, UMAP plots of combined (*left panel*), control (*middle panel*), and *Pramel12* KO (*right panel*) spermatids revealing four distinct subcell types: Early RS, early round spermatids; Middle-late RS, middle to late stage round spermatids; Early elongating, early elongating spermatids; Elongated, mature elongated spermatids. *B*, dot plot for the selected marker genes across the four identified subcell types. *C*, summary of detailed cell numbers and percentages of spermatids in each subcell type in control and *Pramel12* mutant testes. *D*, volcano plots of DEGs in the four subcell types of *Pramel12* KO testes using a cutoff of *p* < 0.05 and |log_2_ fold change| >0.2. *E* and *F*, Venn diagrams of upregulated genes (*E*) and downregulated genes (*F*) among the four subcell types. DEG, differentially expressed gene; scRNA-Seq, single-cell RNA-Seq; UMAP, Uniform Manifold Approximation and Projection.

Given the defects in spermiogenesis in *Pramel12*-null mice, spermatids and elongating spermatozoa were isolated and reclustered. A total of 10,903 control cells and 9437 *Pramel12*-null cells were analyzed. Based on UMAP and marker gene analyses, four distinct cell subtypes were identified (Fig. 6, *A* and *B*). Cluster identities were assigned according to the expression of spermatid and spermatozoa marker genes. Cluster 1 cells exhibited predominant expression of *Ccna1* and *Tex36*, classifying them as early RS (steps 1–4). Cluster 2 cells expressed high levels of *Ccna1*, *Tex36*, and *Sun5*, consistent with middle-late RS (steps 5–8). Cluster 3 cells corresponded to early elongating spermatozoa (early elongating, steps 9–12), as they expressed high levels of *Prm1* and low levels of *Cstl1*. Cluster 4 cells expressed high levels of *Prm1* and *Cstl1*, identifying them as elongated spermatozoa (elongated, steps 13–16). These expression patterns of spermatid marker genes recapitulated the developmental sequence of spermiogenesis, from early RS to elongated spermatozoa.

In control samples, 28.77%, 20.30%, 23.49%, and 27.44% of cells were sorted into early RS, middle-late RS, early elongating, and elongated subtypes, respectively. In *Pramel12*-null samples, 26.77%, 18.29%, 23.88%, and 31.06% of cells were sorted into these respective subtypes (Fig. 6*C*).

### *Pramel12* deficiency disrupts multistage spermiogenic regulators

**Figure 7:**
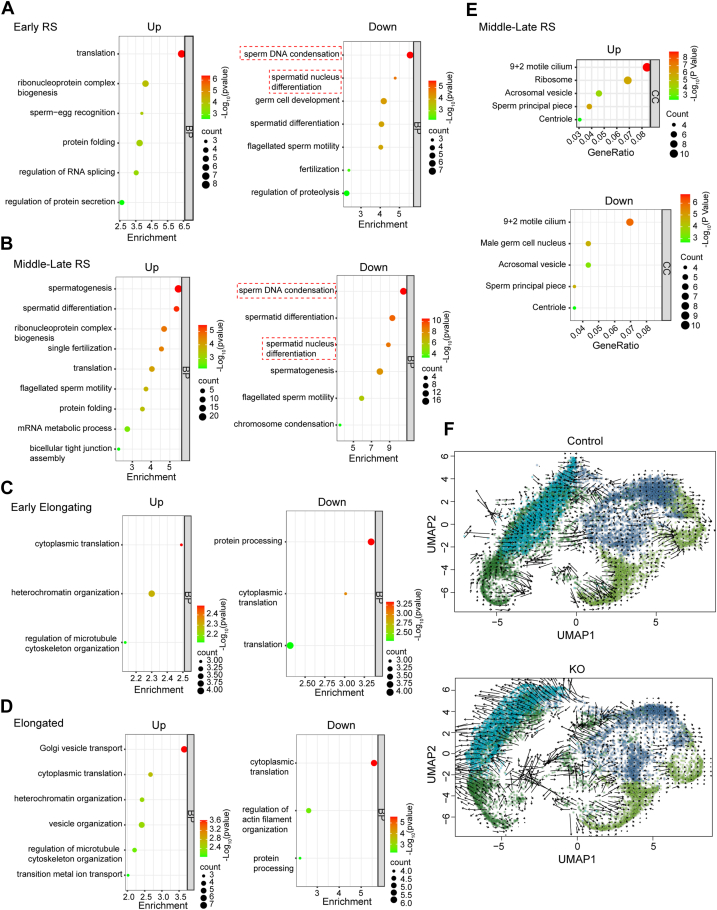
**GO analysis and RNA velocity dynamics of *Pramel12*-null spermatids.***A*, GO terms of biological processes for upregulated (*left panel*) and downregulated (*right panel*) genes in *Pramel12*-null early RS. *B*, same as (*A*), but in *Pramel12*-null middle-late RS. *C*, same as (*A*), but in *Pramel12*-null early elongating. *D*, same as (*A*), but in *Pramel12*-null elongated. *E*, GO analysis of cellular components for upregulated and downregulated transcripts in *Pramel12*-null middle-late RS. *F*, RNA velocity dynamics in control and *Pramel12* KO samples across the four spermatid subcell types. RNA velocities were visualized on the existing embedding UMAP. GO, Gene Ontology; RS, round spermatid; UMAP, Uniform Manifold Approximation and Projection.

Differential gene expression analyses were performed for each cell subtype using a threshold of *p* < 0.05 and |log_2_ fold change| >0.2. Volcano plots revealed distinct patterns of differentially expressed genes (DEGs) across the cell subtypes. In early RS, 111 DEGs were identified (50 upregulated, 61 downregulated), in middle-late RS, 273 DEGs were identified (146 upregulated, 127 downregulated), in early elongating spermatozoa, 148 DEGs were identified (79 upregulated, 69 downregulated), and in elongated spermatozoa, 289 DEGs were identified (138 upregulated, 151 downregulated) (Fig. 6*D*). The majority of DEGs identified in early RS and early elongating spermatids persisted into their respective later developmental stages, middle-late RS, and elongated spermatids (Fig. 6, *E* and *F*, *left panels*). Notably, there was significant overlap in DEGs across the four cell subtypes (Fig. 6, *E* and *F*, *right panels*). In early RS, Gene Ontology (GO) term analysis showed an enrichment of terms for translation and ribonucleoprotein complex biogenesis in *Pramel12*-null mice, whereas terms related to sperm DNA condensation, spermatid nucleus differentiation, sperm motility, and fertilization were reduced (Fig. 7*A*). In middle-late RS, upregulated genes were associated with spermatid differentiation, translation, sperm motility, and fertilization, whereas downregulated genes were linked to sperm DNA condensation, spermatid nucleus differentiation, spermatogenesis, and sperm motility (Fig. 7*B*). Importantly, biological processes related to sperm DNA condensation and spermatid nucleus differentiation were significantly reduced in both early and middle-late RS, consistent with a severe impairment in the final stages of sperm maturation in *Pramel12*-null mice. GO term analyses of DEGs in early elongating and elongated spermatids revealed significant disturbances in processes, such as cytoplasmic translation, regulation of microtubule cytoskeleton organization, and protein processing in *Pramel12*-null mice (Fig. 7, *C* and *D*). In addition, GO terms for cellular components in middle-late RS were significantly enriched in "9 + 2" motile cilium, acrosomal vesicle, sperm principal piece, and centriole (Fig. 7*E*), consistent with the observed abnormalities in *Pramel12*-deficient sperm.

To further investigate these observations, RNA velocity analysis was performed across the four cell subtypes. RNA velocity predicts the future transcriptional states of individual cells, thereby inferring transcriptional and differentiation trajectories (40). RNA velocity dynamics were comparable between control and *Pramel12*-null samples in both early and middle-late RSs but became divergent in early elongating and elongated spermatids. In the control sample, early elongating cells exhibited a clear, smooth progression from early elongating to elongated cells, with elongated cells showing terminal differentiation points on the UMAP plot (Fig. 7*F*), indicating normal differentiation into the final stage. In contrast, early elongating cells in *Pramel12*-null samples exhibited disorganized transcriptional states, with weak progression toward elongated cells. The velocity vectors were dispersed in different directions, with fewer vectors pointing toward elongated cells (Fig. 7*F*). In addition, cells in the elongated cluster did not display terminal differentiation points on the UMAP but instead exhibited long velocity vectors pointing in various directions (Fig. 7*F*), suggesting disrupted differentiation. This analysis indicates that *Pramel12* deficiency impairs spermatid differentiation and maturation.

To validate the transcriptional dysregulation, an RT–qPCR assay was conducted on spermiogenic genes in *Pramel12*-null testes. Genes involved in sperm chromatin condensation and protamination (*e.g.*, *Tnp1*, *Tnp2*, *Prm1*, *Prm2*, *Tssk6*), sperm deformation and motility (*e.g.*, *Spem1*, *Galntl5*), acrosome function and fertilization (*e.g.*, *Iqcf1*, *Lyzl4*), mitochondrial function (*e.g.*, *Spata19*), flagella integrity (*e.g.*, *Cabs1*, *Ropn1*), and sperm morphology and maturation (*e.g.*, *Fhl5*) exhibited significantly decreased expression in *Pramel12*-null testes (Fig. S12, *A*–*M*), consistent with the observed defects in *Pramel12*-deficient sperm. These results collectively demonstrate that *Pramel12* deficiency leads to dysregulation of spermiogenic genes critical for multiple stages of spermiogenesis, ultimately disrupting spermatid differentiation and maturation.

### *Pramel12* deficiency perturbs chromatin remodeling during spermiogenesis

**Figure 8:**
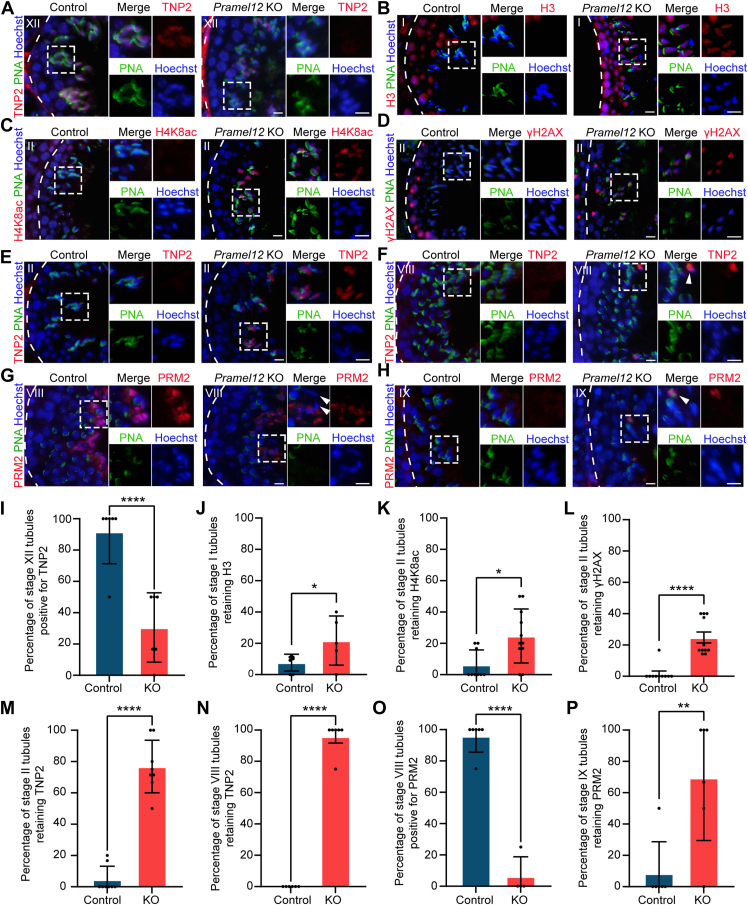
***Pramel12* deficiency perturbs chromatin remodeling during spermiogenesis.***A*–*H*, immunofluorescence analyses of testicular sections from adult control and *Pramel12*-null mice with the following combinations: (*A*) PNA and TNP2; (*B*) PNA and H3; (*C*) PNA and H4K8ac; (*D*) PNA and γH2AX; (*E*) PNA and TNP2; (*F*) PNA and TNP2; (*G*) PNA and PRM2; and (*H*) PNA and PRM2. DNA was stained with Hoechst 33342. The scale bar represents 10 μm. *I*–*P*, statistical analyses of immunofluorescence data on chromatin remodeling factors. *I*, percentage of stage XII tubules positive for TNP2 in control and *Pramel12*-null testes. *J*, percentage of stage I tubules retaining H3. *K*, percentage of H4K8ac retention in stage II tubules. *L*, percentage of stage II tubules retaining γH2AX. *M*, percentage of TNP2 retention in stage II tubules. *N*, percentage of TNP2 retention in stage VIII tubules. *O*, percentage of stage VIII tubules positive for PRM2. *P*, percentage of PRM2 retention in stage IX tubules. Data are presented as mean ± SD; ∗*p* < 0.05, ∗∗*p* < 0.01, and ∗∗∗∗*p* < 0.0001. PNA, peanut agglutinin; PRM, protamine; TNP, transition protein.

One of the critical processes during spermiogenesis is histone-to-PRM chromatin remodeling, wherein transition proteins (TNPs) replace histones before being further substituted by sperm-specific PRMs, facilitating the repackaging of the genome into a highly compact chromatin structure (41, 42, 43). Hyperacetylation of histones is commonly observed in stages IX to XI tubules prior to the replacement of histones by TNPs, with this modification gradually disappearing as TNPs take over (44, 45, 46). The processes of sperm DNA condensation and spermatid nuclear differentiation were significantly reduced in *Pramel12*-null spermatids, indicating disrupted chromatin remodeling. To examine these defects, immunostaining was performed using antibodies against H3, H4K8ac, TNP2, and PRM2. Immunofluorescence staining showed similar expression patterns of H3 and H4K8ac in step 9 spermatids within stage IX tubules of both control and *Pramel12*-null testes (Fig. S13, *A* and *B*). In control testes, TNP2 signals were detected in step 12 spermatids at stage XII tubules, confirming successful histone replacement. In contrast, *Pramel12*-null spermatids exhibited markedly diminished TNP2 signals, indicating impaired histone displacement (Fig. 8, *A* and *I*). Notably, in stage I tubules, H3 signals largely disappeared in step 13 spermatids of control testes, but substantial retention was observed in *Pramel12*-null testes (Fig. 8, *B* and *J*). Similarly, H4K8ac signals were almost undetectable in step 14 spermatids within stage II tubules in control testes, whereas *Pramel12*-null spermatids exhibited persistent H4K8ac labeling in a greater proportion of stage II tubules (Fig. 8, *C* and *K*). Thus, defective clearance of H3 and H4K8ac prevents the proper incorporation of TNP2 in *Pramel12*-deficient spermatids.

Transient DNA strand breaks in elongating spermatids are essential for DNA relaxation and subsequent chromatin compaction during spermiogenesis (47). These transient endogenous nicks are required to eliminate free DNA supercoils resulting from histone withdrawal (48), and the absence of DNA strand breaks leads to low chromatin compaction (49). Comparable levels of DNA strand breaks, marked by phosphorylated H2AX (γH2AX), were observed in step 9 spermatids at stage IX tubules in both control and *Pramel12*-null testes (Fig. S13*C*). However, γH2AX signals persisted in step 14 spermatids, with an increased frequency observed in stage II tubules of *Pramel12*-null testes, whereas DNA repair was largely completed by the same spermiogenic step in control testes (Fig. 8, *D* and *L*). These results indicate that transient DNA strand breaks and repair processes are disturbed in *Pramel12*-deficient spermatids.

PRM2, first detected at step 14 and retained in mature sperm (50), was examined for its role in completing TNP2 replacement in elongated spermatids (steps 14–16). Immunofluorescence for TNP2 revealed successful clearance in elongated spermatids of control stage II/VIII tubules, whereas *Pramel12*-null testes exhibited persistent TNP2 retention in these spermatids at higher frequencies (Fig. 8, *E*, *F*, *M* and *N*). Furthermore, PRM2 incorporation was observed in nearly all control step 16 spermatids at stage VIII, whereas only a small percentage of stage VIII tubules from *Pramel12*-null testes contained PRM2-positive spermatids, indicating a defective histone–PRM transition (Fig. 8, *G* and *O*). Increased PRM2-positive spermatids were detected in stage IX tubules of *Pramel12*-null testes (Fig. 8, *H* and *P*), correlating with spermiation failure. These results suggest that *Pramel12* deficiency disrupts spermiogenic chromatin remodeling by impairing the histone-to-PRM transition.

To investigate the fate of spermatids with persistent γH2AX, we assessed apoptosis across steps 9 to 16. A significantly higher percentage of tubules in *Pramel12*-null testes contained TUNEL-positive spermatids compared with controls (Fig. S14, *A* and *B*). In these mutants, unreleased spermatids showed TUNEL signals, and γH2AX-positive spermatids colocalized with TUNEL staining (Fig. S14*C*), indicating defective DNA repair. Apoptosis occurred throughout steps 9 to 16 but was significantly more frequent at steps 13 to 14 (Fig. S14*D*). These results indicate that defective nuclear condensation during spermiogenesis steps 13 to 14 primarily induces apoptosis in *Pramel12*-deficient testes.

### Proteomic profiling of *Pramel12*-null testes

**Figure 9:**
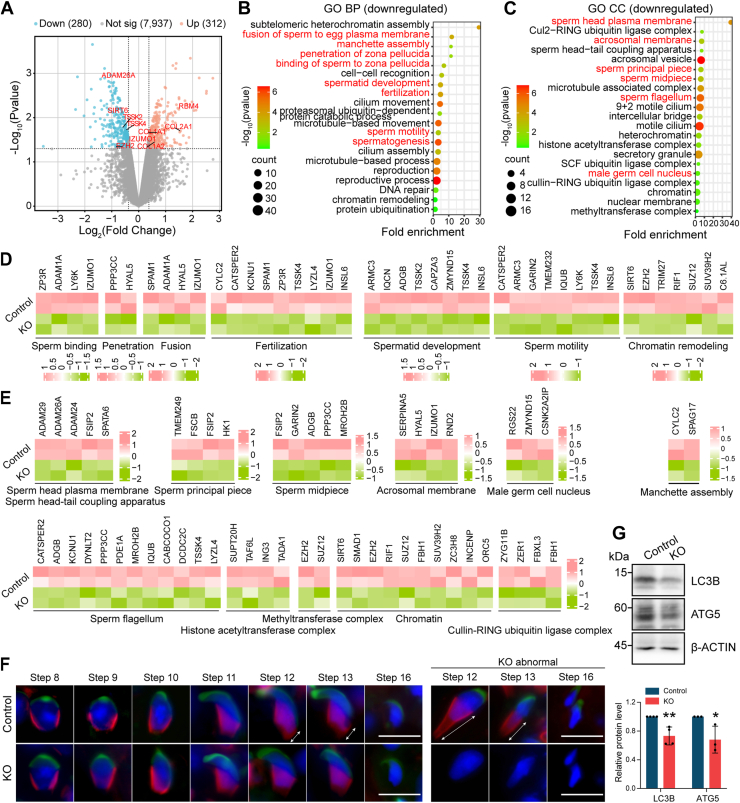
**Proteomic analysis of *Pramel12*-null testes.***A*, volcano plot of differentially expressed proteins. *B*, biological process GO terms for downregulated proteins. *C*, cellular component GO terms for downregulated proteins. *D* and *E*, heatmaps displaying differentially expressed proteins corresponding to GO terms in (*B*) and (*C*), respectively. *F*, PNA and α-TUBULIN coimmunostaining in steps 8 to 16 spermatids from control and *Pramel12*-null mice. Nuclei counterstained with Hoechst 33342. The scale bar represents 10 μm. *G*, *upper panel*, immunoblots of LC3B and ATG5 (β-actin loading control). *Lower panel*, relative LC3B and ATG5 protein abundance. Data are presented as mean ± SD; ∗*p* < 0.05, ∗∗*p* < 0.01. GO, Gene Ontology; PNA, peanut agglutinin.

To assess the impact of *Pramel12* depletion on spermatogenesis at the protein level, we performed proteomic analysis on the testes from 4-month-old control and *Pramel12*-null mice. Principal component analysis (PCA) and heatmaps showed strong consistency within replicates and clear differential expression between conditions (Fig. S15, *A* and *B*). Volcano plot analysis identified 312 significantly upregulated and 280 downregulated proteins in *Pramel12*-null testes (Fig. 9*A*). GO analysis indicated that biological processes, such as sperm binding, motility, fertilization, spermatid development, chromatin remodeling, and manchette assembly, were significantly downregulated in *Pramel12*-null testes (Fig. 9, *B* and *D*). Conversely, terms involved in stress-responsive translational regulation, actin-filament bundle assembly, and extrinsic apoptotic signaling pathways were upregulated (Fig. S15, *C* and *D*). Cellular component GO analysis showed that downregulated proteins were enriched in sperm structures (*e.g.*, head plasma membrane, principal piece, midpiece, flagellum, acrosome membrane), the male germ cell nucleus and chromatin, and protein complexes (*e.g.*, methyltransferase, histone acetyltransferase, and cullin–RING ubiquitin ligase complexes) (Fig. 9, *C* and *E*). In contrast, upregulated proteins were associated with the actin cytoskeletal and extracellular matrix (Fig. S15, *E* and *F*). These proteomic findings confirm the previously documented defects in sperm structure and function. The manchette, a transient microtubule scaffold, is essential for spermatid nuclear elongation and chromatin condensation (51). Consistent with reduced manchette assembly, the manchette elongated abnormally in condensing spermatids (steps 12–13) of *Pramel12*-deficient testes (Fig. 9*F*). Considering the critical role of autophagy core protein ATG5 in male fertility and spermiogenesis (52), ATG5 and LC3B levels were examined in *Pramel12*-null testes. The results revealed significant reductions in both ATG5 and LC3B abundance (Fig. 9*G*), suggesting impaired autophagic function. Proteomic analysis thus indicates that *Pramel12* deficiency leads to substantial dysregulation of proteins involved in spermatid structure, function, and protein modification.

### Protein profile alterations in *Pramel12*-deficient testes and sperm

**Figure 10:**
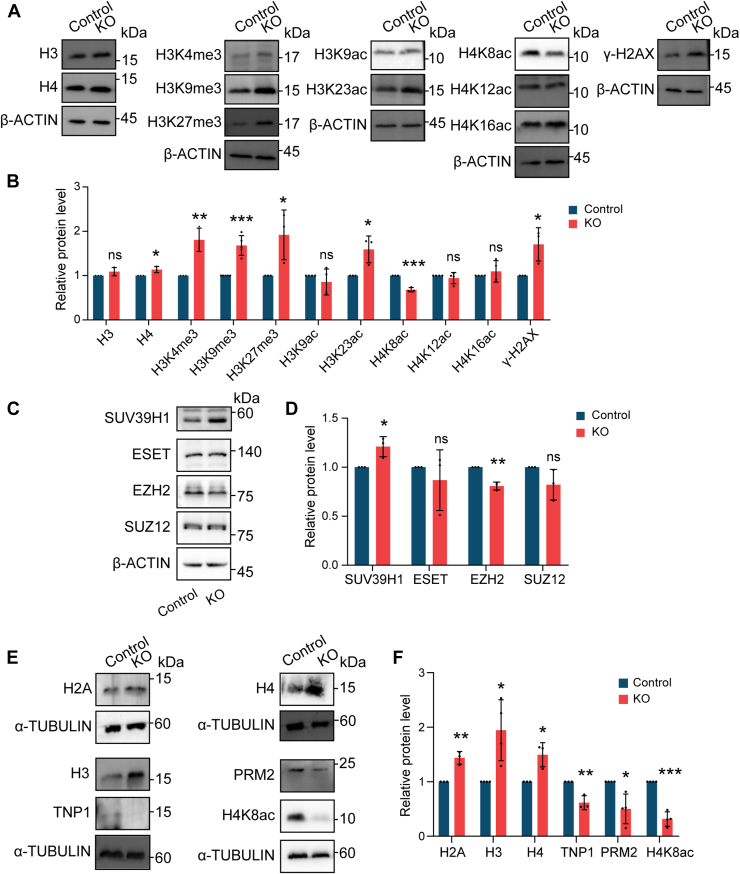
**Protein abundance analysis in *Pramel12*-deficient testes and spermatozoa.***A*, immunoblot analysis of core histones (H3, H4), histone PTMs (methylation: H3K4me3, H3K9me3, and H3K27me3; acetylation: H3K9ac, H3K23ac, H4K8ac, H4K12ac, and H4K16ac), and γ-H2AX in 4-month-old control and *Pramel12*-null testes. *B*, quantification of protein abundance shown in (*A*). *C*, immunoblots of SUV39H1, ESET, EZH2, and SUZ12 (β-actin loading control) in adult control and *Pramel12*-null testes. *D*, quantified relative abundance of proteins in (*C*). *E*, immunoblot analysis of H2A, H3, H4, TNP1, PRM2, and H4K8ac (α-TUBULIN loading control) in control and *Pramel12*-deficient sperm. *F*, relative protein abundance derived from (*E*). Data are presented as mean ± SD; ns, no significance; ∗*p* < 0.05, ∗∗*p* < 0.01, and ∗∗∗*p* < 0.001. PTM, post-translational modification.

Histone post-translational modifications (PTMs) play a key role in chromatin loosening during spermiogenesis. In mice, histone methylation and acetylation mark early elongated spermatids but are nearly absent in late stages, coinciding with histone-to-PRM replacement (53). To further investigate chromatin remodeling, this study profiled core histones (H3, H4), histone PTMs (methylation: H3K4me3, H3K9me3, H3K27me3; acetylation: H3K9ac, H3K23ac, H4K8ac, H4K12ac, H4K16ac), and γ-H2AX in control *versus Pramel12*-null adult testes. Quantified immunoblots demonstrated significantly elevated core histone methylation (K4me3, K9me3, and K27me3) and increased H3K23ac but decreased H4K8ac acetylation in *Pramel12*-null testes (Fig. 10, *A* and *B*), suggesting aberrant histone modifications upon *Pramel12* deletion. In addition, γ-H2AX levels were significantly increased (Fig. 10, *A* and *B*), possibly indicating increased apoptosis or insufficient DNA strand break repair during spermiogenesis. Further analysis of histone methylation enzymes showed an increase in SUV39H1, a decrease in EZH2, and no significant change in ESET or SUZ12 in *Pramel12*-null testes (Fig. 10, *C* and *D*), implying that the aberrant histone modification may be attributable to a perturbation of methyltransferase activity upon *Pramel12* loss.

Finally, the impact of altered histone modifications on TNP1 and PRM2 integration and histone displacement in *Pramel12*-deficient sperm was examined. Immunoblot analysis demonstrated that sperm from *Pramel12*-null mice contained elevated levels of histones H2A, H3, and H4, alongside decreased levels of H4K8ac, TNP1, and PRM2 (Fig. 10, *E* and *F*), suggesting increased retention of histones and impaired incorporation of TNPs and PRMs. These results establish that PRAMEL12 is essential for the histone-to-PRM transition during spermiogenesis in mice.

### Model of PRAMEL12 in spermatogenesis

**Figure 11:**
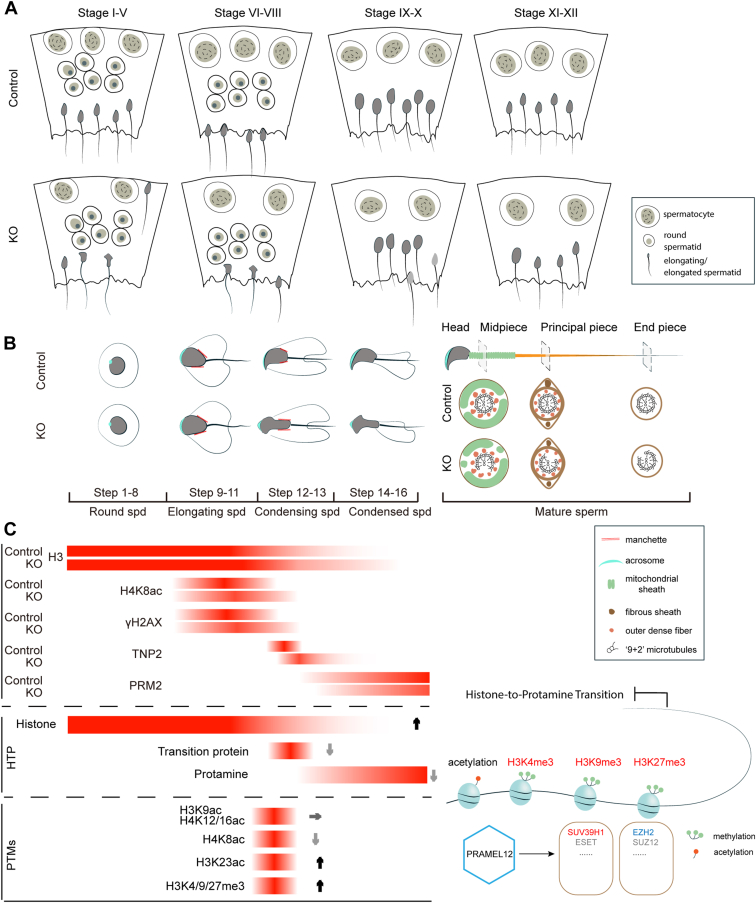
**Proposed model of PRAMEL12 during spermatogenesis.***A*, schematic diagram of spermatogenic phenotypes in seminiferous tubules of *Pramel12*-null mice. *B*, schematic diagram of sperm head malformations during the condensation phase of spermiogenesis (*left panel*) and disrupted flagellar axoneme structure (*right panel*) in *Pramel12*-deficient mice. *C*, schematic diagram depicting expression patterns of H3, H4K8ac, γH2AX, TNP2, and PRM2, alongside histone modification dynamics and the histone-to-protamine transition during spermiogenesis. PRAME, preferentially expressed antigen of melanoma; PRM, protamine.

*Pramel12* is crucial for mouse spermatogenesis and male fertility. Its loss disrupts the seminiferous epithelium cycle and spermiogenesis, leading to reduced sperm counts, decreased motility, increased sperm malformations, and infertility (Fig. 11*A*). *Pramel12* deficiency impairs spermatid chromatin condensation and nuclear maturation, resulting in sperm head morphological defects, compromised mitochondrial integrity, and disorganized axoneme structure (Fig. 11*B*). Deletion of *Pramel12* causes aberrant histone modifications and disrupts methyltransferase activity. Ultimately, *Pramel12* ablation leads to increased histone retention and decreased integration of TNPs and PRMs, disrupting the histone-to-PRM transition (Fig. 11*C*).

## Discussion

PRAME family members are widely studied in cancer because of their high expression in many malignancies. They are also found in testicular and ovarian tissues, but their roles in gametogenesis and reproduction are still not well understood. PRAMEX1 and PRAMEL1 have been shown to cooperatively suppress the RA signaling pathway, thereby maintaining normal spermatogonial homeostasis and spermatogenesis in male mice. KO of either *Pramex1* or *Pramel1*, as well as their double KO, results in a region-specific Sertoli cell–only phenotype and impaired spermatogenesis (29, 30, 31). PRAMEF12 is essential for spermatogonial stem cell self-renewal and proliferation; disruption of *Pramef12* impairs spermatogonial maintenance, halts spermatogenesis, and leads to a Sertoli cell–only phenotype and male sterility (32). While specific PRAME family members are well-documented regulators of spermatogonial maintenance, our study reveals a previously uncharacterized role for PRAMEL12 in spermiogenesis. Using an integrated multiomic approach, we demonstrate that PRAMEL12 is crucial for this process. Mechanistically, PRAMEL12 deficiency disrupts the histone-to-PRM transition, leading to aberrant sperm morphology and male infertility.

Although our study employed a cross-sectional design with two primary timepoints (4 and 12 months), the stark contrast between these ages provides compelling evidence for a time-dependent phenotype. The absence of major morphological defects in young adult (4-month) *Pramel12*-null testes, apart from spermiogenesis-specific errors, indicates that the initial establishment of spermatogenesis is largely intact. However, the cumulative impact of these errors, such as impaired spermiation, loss of sperm individualization, defective chromatin remodeling, and increased apoptosis, appears to overwhelm the system over time. This ultimately manifests as a catastrophic failure by 12 months, characterized by significant testicular atrophy, a drastic loss of meiotic and postmeiotic cells, and a near-complete absence of sperm. Future longitudinal studies tracking the same cohort of mice over time would be valuable to precisely define the kinetics of this decline.

Emerging evidence from the expression patterns of PRAME family members highlights their essential roles across the mitotic, meiotic, and postmeiotic phases of spermatogenesis. PRAMEF12 is specifically expressed in mouse spermatogonia (32). PRAMEL1 and PRAMEX1 localize to various cellular organelles at different stages of spermatogenesis, including the nucleus, rough endoplasmic reticulum, Golgi, mitochondria, intermitochondrial cement, chromatoid body, centrioles, manchette, and flagellum (27). In bovines, Y-linked PRAME (PRAMEY) is expressed in the nucleus and several cytoplasmic organelles, including the rough endoplasmic reticulum, Golgi vesicles, intermitochondrial cement, chromatoid body, centrioles, acrosome of the sperm head, and the middle piece of the sperm tail (25, 26). Although *Pramel12* transcripts are highly expressed in spermatocytes and spermatids, we cannot exclude the possibility that the observed phenotype originates from Sertoli cells. In addition, the deletion of exons 3 through part of exon 5 *via* CRISPR technology does not rule out potential contributions from off-target effects or strain-specific background variants. Therefore, a conditional KO model of *Pramel12* in germ cells would be valuable to further verify its role in spermiogenesis.

Spermiogenesis, the terminal phase of spermatogenesis involving sperm cytoplasmic remodeling and nuclear compaction, is essential for producing structurally and functionally intact spermatozoa. Impairments in this process can lead to teratozoospermia (abnormal sperm morphology), oligozoospermia (reduced sperm count), and asthenozoospermia (impaired sperm motility). Our findings reveal defects in sperm head morphology, impaired spermiation, and loss of individualization. These phenotypic abnormalities are consistently reflected at the transcriptional level. The emergence of head malformations during the nuclear condensation phase is clearly captured by our scRNA-Seq data, which demonstrate a significant disruption in the differentiation trajectory of elongating spermatids, along with specific downregulation of genes associated with sperm DNA condensation and spermatid nucleus differentiation within the spermatid cluster.

Immunofluorescent analysis of H3, H4K8ac, γH2AX, TNP2, and PRM2 revealed severe disruption of the histone-to-PRM transition in the absence of PRAMEL12. This impairment, clearly observable *via* immunofluorescence, was further corroborated mechanistically by proteomic analysis, which identified significant dysregulation among proteins involved in chromatin remodeling and methyltransferase–histone acetyltransferase complexes. Together, these findings indicate that PRAMEL12 plays an essential role in chromatin remodeling during spermiogenesis.

Proteomic analysis identified differentially abundant proteins related to spermatid structure, function, and protein modification. Histone PTMs are crucial for chromatin remodeling and subsequent histone displacement during spermiogenesis (53). Increased histone retention and altered PRM levels in spermatids and sperm have been strongly associated with male infertility in mice (54). *Pramel12*-null testes exhibited significant aberrations in histone PTMs. Furthermore, sperm lacking *Pramel12* displayed elevated levels of histones H2A, H3, and H4, accompanied by reduced levels of TNP1 and PRM2. These molecular alterations indicate severely compromised histone displacement and a defective histone-to-PRM transition. Consequently, these defects directly manifest as poor sperm quality, characterized by aberrant chromatin compaction (which underlies the observed head morphology defects) and functional incompetence (accounting for the reduced motility and infertility).

The IVF assay revealed impaired fertilization capacity of *Pramel12*-null sperm, as evidenced by a significantly lower cleavage rate. Moreover, none of the resulting two-cell embryos in the null group developed to the blastocyst stage, suggesting compromised developmental potential. However, we cannot exclude the possibility that the absence of blastocyst formation was due to the low number of two-cell embryos available for analysis. Further investigation using a larger cohort of two-cell embryos or intracytoplasmic sperm injection assays is needed to conclusively determine the embryo developmental potential. Although the IVF assay was normalized for total sperm concentration, we cannot formally rule out that the observed fertilization defect in *Pramel12*-null males stems partly from a reduced proportion of competent sperm, given their drastically impaired motility and morphology. Nevertheless, our multiomics and ultrastructural data suggest that the failure is fundamentally because of a cell-autonomous functional impairment. The profound dysregulation of fertilization-critical genes and proteins, coupled with severe structural defects and a premature acrosome reaction, indicates that *Pramel12* ablation produces a globally incompetent sperm population. Thus, the IVF results likely reflect an intrinsic functional deficit in the null sperm themselves, rather than merely an artifact of population normalization. Furthermore, the *in vivo* mating assay indicated that no fertilized embryos were obtained from *Pramel12*-null males. This could be due to severely impaired sperm motility, potentially preventing sperm from traversing the female genital tract and uterus to reach the ampulla for fertilization.

A recent study identified PRAME as a substrate-recognition subunit within E3 ubiquitin ligase complexes, where it directs specific proteins for ubiquitination and subsequent proteasomal degradation. In multiple myeloma cells, PRAME facilitates cell proliferation by recognizing CTMP and P21 through a CUL2-dependent mechanism, promoting their ubiquitination and degradation (55). Similarly, PRAMEL7 recruits CUL2 to mediate the degradation of UHRF1 and the NuRD complex, establishing ground-state pluripotency *via* integrated proteasomal and epigenetic pathways (56, 57). PRAMEL15 promotes DNA methylation reprogramming in early mouse embryogenesis by mediating the Cullin–RING E3 ligase–dependent degradation of DNMT1 (58). These findings suggest that PRAME family members may act as substrate-binding subunits of E3 ubiquitin ligase complexes within the ubiquitin–proteasome system. However, the potential role of PRAMEL12 in the ubiquitin–proteasome pathway during spermiogenesis remains an area for further investigation.

In summary, using a *Pramel12* KO model, our findings establish PRAMEL12, a member of the cancer/testis antigen family, as a newly identified and essential regulator of spermiogenesis and male fertility. Global deletion of *Pramel12* causes severe sperm malformations because of defects in the histone-to-PRM transition. Our work not only provides genetic evidence linking impaired PRAMEL12-dependent chromatin dynamics to OAT pathogenesis but also broadens the functional repertoire of PRAME family proteins beyond cancer biology to include reproductive health.

## Experimental procedures

### Animals

All animal procedures were conducted following the guidelines approved by the Ethics Committee for Animal Research of the School of Life Sciences, Shandong University, China (approval no.: SYDWLL-2025-005). The *Pramel12* mutant mice were purchased from Cyagen. To obtain *Pramel12*^−/−^ (*Pramel12* KO) males, we crossed *Pramel12*^+/−^ males with fertile *Pramel12*^−/−^ females. The resulting *Pramel12*^+/−^ males served as controls in all subsequent experiments. Genotyping primers are listed in Table S1.

### Fertility assay

To assess male and female fertility, we performed continuous mating over 6 months. Wildtype females were paired with control or *Pramel12*-null males, and wildtype males were paired with control or *Pramel12*-null females. The average litter size was recorded from at least three independent mating cages per genotype.

### Histology analysis

Mouse testes and epididymides were fixed in Bouin’s solution overnight at 4 °C, followed by standard dehydration and paraffin embedding. Sections were cut to 5 μm, stained with H&E, and images were acquired using a Nexcope NE950 microscope. Sperms collected from the cauda epididymides were spread onto slides, air-dried, fixed with 4% paraformaldehyde (PFA) for 30 min, and stained with H&E. Images were captured using the same microscope.

### *In* situ hybridization

The sequence of the mouse *Pramel12* probe for *in situ* hybridization was retrieved from the GenePaint database. Templates were generated *via* PCR from *Pramel12* complementary DNA (cDNA) and subcloned into the pEASY Blunt Zero cloning vector. The construct was linearized and used as a template for *in vitro* transcription with T7 polymerase to generate a probe labeled with digoxigenin-11-UTP. *In situ* hybridization analysis was performed as described previously (59). Signals were developed in NBT/BCIP stock solution and captured with an upright microscope (Nexcope NE950).

### Immunofluorescence

Sections were dewaxed, rehydrated, and subjected to antigen retrieval with 0.01% sodium citrate buffer (pH 6.0). Sections were then blocked in blocking buffer at room temperature for 1 h, followed by overnight incubation with primary antibodies (listed in Table S2) at 4 °C. Secondary antibodies (Table S2) were incubated at room temperature for 1 h the next day. DNA was stained with Hoechst 33342. The sections were mounted, and images were captured using a Nexcope NE950 fluorescence microscope.

### Meiotic chromosome spreads

To isolate germ cells, the tunica albuginea was first removed. Seminiferous tubules were then incubated for 30 min in hypotonic buffer (30 mM Tris [pH 7.5], 50 mM sucrose, 17 mM trisodium citrate, and 5 mM EDTA). Subsequently, tubules were gently disrupted in 200 mM sucrose to release germ cells. Following treatment with fixation buffer (1% PFA and 0.1% Triton X-100 in PBS), cells were spread onto slides and air-dried.

### TUNEL assay

For TUNEL assay, testicular sections were dewaxed and treated with proteinase K for 30 min at room temperature. Sections were incubated with TdT enzyme and fluorescent solution at 37 °C for 1 h. DNA was stained with Hoechst 33342, and fluorescent images were captured using a Nexcope NE950 fluorescence microscope, following the protocol from the staining kit (Beyotime, C1088).

### Immunoblot assay

Total protein was extracted using radioimmunoprecipitation assay lysis buffer. Proteins were separated by SDS-PAGE, followed by electrophoretic transfer onto polyvinylidene fluoride membranes. Membranes were blocked with 5% nonfat milk at room temperature for 1 h, incubated with primary antibodies (listed in Table S2) overnight at 4 °C, and then incubated with secondary antibodies (Table S2) at room temperature for 1 h. Protein signals were developed using ECL substrate (Biosharp Biological Technology Co, Ltd, BL523B) according to the manufacturer’s protocol. Uncropped immunoblot images are presented in Figs. S16 and S17.

### RNA isolation and RT–qPCR

Total RNA was extracted using the AFTSpin Tissue/Cell Fast RNA Extraction Kit for Animal (ABclonal, RK30120), and cDNA synthesis was performed with the ABScript III RT Master Mix (ABclonal, RK20429). RT–qPCR was performed on the Q2000B Real-Time qPCR System using the 2× Universal SYBR Green Fast qPCR Mix (ABclonal, RK21203). Primers used are listed in Table S3. The relative abundance of each transcript was calculated using the 2^−ΔΔCt^ method, with normalization to the endogenous β-actin control (60).

### Spontaneous acrosome reaction

Sperms isolated from the cauda epididymides were incubated in TYH medium at 37 °C in 5% CO_2_ for either 0 h (noncapacitated) or 1 h (capacitated). Both noncapacitated and capacitated sperms were spread onto slides, air-dried, fixed with 4% PFA, and stained with FITC-conjugated *Arachis hypogaea* (peanut) PNA (15 μg/ml) at 37 °C for 1 h. Sperm nuclei were stained with Hoechst 33342, and fluorescent images were captured using a Nexcope NE950 fluorescence microscope. The spontaneous acrosome reaction frequency was determined by calculating the ratio of PNA-negative sperm to Hoechst 33342-positive sperm.

### Computer-assisted sperm analysis

Sperm collected from cauda epididymides were released into M2 medium for 10 min at 37 °C in 5% CO_2_. A 10 μl sperm sample was loaded into a glass cell chamber and observed through a 20× objective lens. Sperm parameters were analyzed using computer-assisted sperm analysis. At least 200 sperms per mouse were analyzed, with the experiment repeated three times.

### *Invitro* fertilization

For IVF, sperms from cauda epididymides were incubated in TYH medium (M2036, Nanjing Aibei Biotechnology Co) at 37 °C in 5% CO_2_ for 1 h to induce capacitation. Cumulus–oocyte complexes were harvested from the oviducts of superovulated female mice and placed into HTF medium (M1135, Nanjing Aibei Biotechnology Co). Capacitated sperms (5–15 μl, at a concentration of 1 × 10^6^ cells/ml) were added to the HTF medium and incubated for 6 h at 37 °C in 5% CO_2_. Following the 6-h incubation, sperm–oocyte complexes were washed and cultured in pre-equilibrated KSOM medium (M1435, Nanjing Aibei Biotechnology Co) at 37 °C in 5% CO_2_. The two-cell cleavage rate and blastocyst formation rate were assessed on day 1 and day 3 of culture post-IVF, respectively.

### Transmission electron microscopy

For electron microscopy, testes and sperm were prefixed with 3% glutaraldehyde, followed by postfixation in 1% osmium tetroxide. Samples were dehydrated in a series of acetone solutions and embedded in Epon-812. Semithin sections were stained with methylene blue, and ultrathin sections were cut with a diamond knife, then stained with uranyl acetate and lead citrate. Finally, sections were examined using a JEM-1400-FLASH transmission electron microscope (JEOL).

### scRNA-Seq library preparation

scRNA-Seq libraries were prepared as described previously (59). Briefly, single cells isolated from control and *Pramel12*-null testes were mixed with a suspension containing barcoded beads and unique molecular identifier (UMI) elements. Following partitioning of thousands of cells into nanoliter-scale Gel Bead-In-EMulsions and barcoding, the full-length barcoded cDNA underwent PCR amplification to yield adequate material for library construction. Libraries were prepared through fragmentation, end repair, A-tailing, adaptor ligation, and index PCR. Finally, the cDNA libraries were sequenced on an Illumina NovaSeq 6000 platform at Beijing EasyResearch Technology Co, Ltd.

### scRNA-Seq data analysis

The Cell Ranger software pipeline (version 7.0.1) from 10× Genomics was employed to demultiplex cellular barcodes, map reads to the genome and transcriptome, and down-sample reads as needed to generate normalized aggregate data across samples. This process produced a raw UMI count matrix, which was then converted into a Seurat object using the R package Seurat (version 4.3.0) (61). Cells with fewer than 200 or more than 8000 expressed genes, fewer than 1000 UMIs, or more than 20% mitochondrial-derived UMIs were excluded from the analysis. Library size normalization was performed using the NormalizeData function in Seurat to obtain normalized counts. Specifically, the “LogNormalize” method normalized the gene expression data for each cell by the total expression, scaled it by a factor of 10,000, and log-transformed the results.

Top variable genes across single cells were identified using the method described by previous research (62). The FindVariableGenes function (mean.function = FastExpMean, dispersion.function = FastLogVMR) in Seurat was used to select the most variable genes. PCA was performed to reduce dimensionality using the RunPCA function in Seurat. Graph-based clustering was conducted to group cells according to their gene expression profiles using the FindClusters function in Seurat. Cells were visualized using the 2-dimensional UMAP algorithm in Seurat *via* the RunUMAP function. Marker genes for each cluster were identified using the FindAllMarkers function (test.use = presto) in Seurat. For each cluster, FindAllMarkers was used to identify positive markers in comparison to all other cells.

DEGs were identified using the FindMarkers function (test.use = presto) in Seurat, with thresholds set at *p* < 0.05 and |log_2_ fold change| >0.2. GO enrichment analysis was conducted using R based on the hypergeometric distribution.

### RNA velocity analysis

RNA velocity analysis was conducted using the velocyto.R program (40). Briefly, spliced and unspliced reads were annotated with velocyto.py in conjunction with Cell Ranger, generating BAM files and an accompanying GTF file, which were then saved as .loom files. The .loom files were loaded into R using the read.loom.matrices function to create count tables for spliced and unspliced reads. Cells in the bottom 0.5% of the total unspliced transcript count were filtered out. In addition, genes with an average spliced expression lower than 0.2 or an average unspliced expression lower than 0.05 in at least one cluster were removed. Finally, velocity-vector arrows were projected onto the UMAP plot generated using Seurat.

### Protein extraction, trypsin digestion, and HPLC fractionation

Total proteins were extracted from testicular samples, and protein concentration was determined using the BCA assay. Trypsin solution (trypsin:protein = 1:50) was added to the proteins, and the protein pellets were digested at 37 °C for 16 h. After alkylation, trypsin (trypsin:protein = 1:100) was added again for digestion at 37 °C for 4 h to complete the digestion cycle. The sample was fractionated by high pH reverse-phase HPLC using an Agilent 300 Extend C18 column.

### LC–MS/MS analysis and database search

Chromatographic separation was performed using the nanoflow rate Vanquish Neo system (Thermo Fisher Scientific), and samples separated by HPLC were analyzed by data-independent acquisition (DIA) mass spectrometry using the Astral high-resolution mass spectrometer (Thermo Scientific). DIA data were processed with DIA-NN software. The software parameters were as follows: trypsin was the enzyme used, with a maximum of one missed cleavage site. The fixed modification was Carbamidomethyl (C), and dynamic modifications were set to Oxidation (M) and Acetyl (Protein N-term). Proteins identified through database retrieval were required to pass the filtering criterion of a false discovery rate <1%.

### Bioinformatics analysis

For statistical significance (*p* < 0.05), proteins with fold changes 1.3 were classified as significantly upregulated, whereas those with fold changes <1/1.3 (approximately 0.769) were considered significantly downregulated. GO annotation for the proteome was obtained from the UniProt-GOA database. Proteins were classified according to GO annotation across three categories: biological process, cellular component, and molecular function. The proteomic experiment was conducted by Cosmos Wisdom Biotechnology Co, Ltd.

### Statistical analysis

Data are presented as the mean ± SD. Statistical analyses were performed using a two-tailed Student’s *t* test, with significance defined as ns (no significance), ∗*p* < 0.05, ∗∗*p* < 0.01, ∗∗∗*p* < 0.001, and ∗∗∗∗*p* < 0.0001.

## Data availability

The scRNA-Seq raw sequencing data generated from this study have been deposited in the Sequence Read Archive with the accession number PRJNA1096843. The mass spectrometry proteomics data are available *via* ProteomeXchange with identifier PXD064046.

## Supporting information

This article contains supporting information.

## Conflict of interest

The authors declare that they have no conflicts of interest with the contents of this article.
