# Supplementary material for: PRAMEL12 orchestrates spermiogenesis to ensure male fertility in mice
Source: J Biol Chem. 2026 Mar 20;302(5):111392. doi: 10.1016/j.jbc.2026.111392 (PMC13092047; doi:10.1016/j.jbc.2026.111392)
Supplement: Supplementary Figures and Table [file mmc1.docx]

**Supporting Information**

**PRAMEL12 orchestrates spermiogenesis to ensure male fertility in mice**

Nana Li^#^, Xiao Wang^#^, Hong Li, Zhengpin Wang*

**
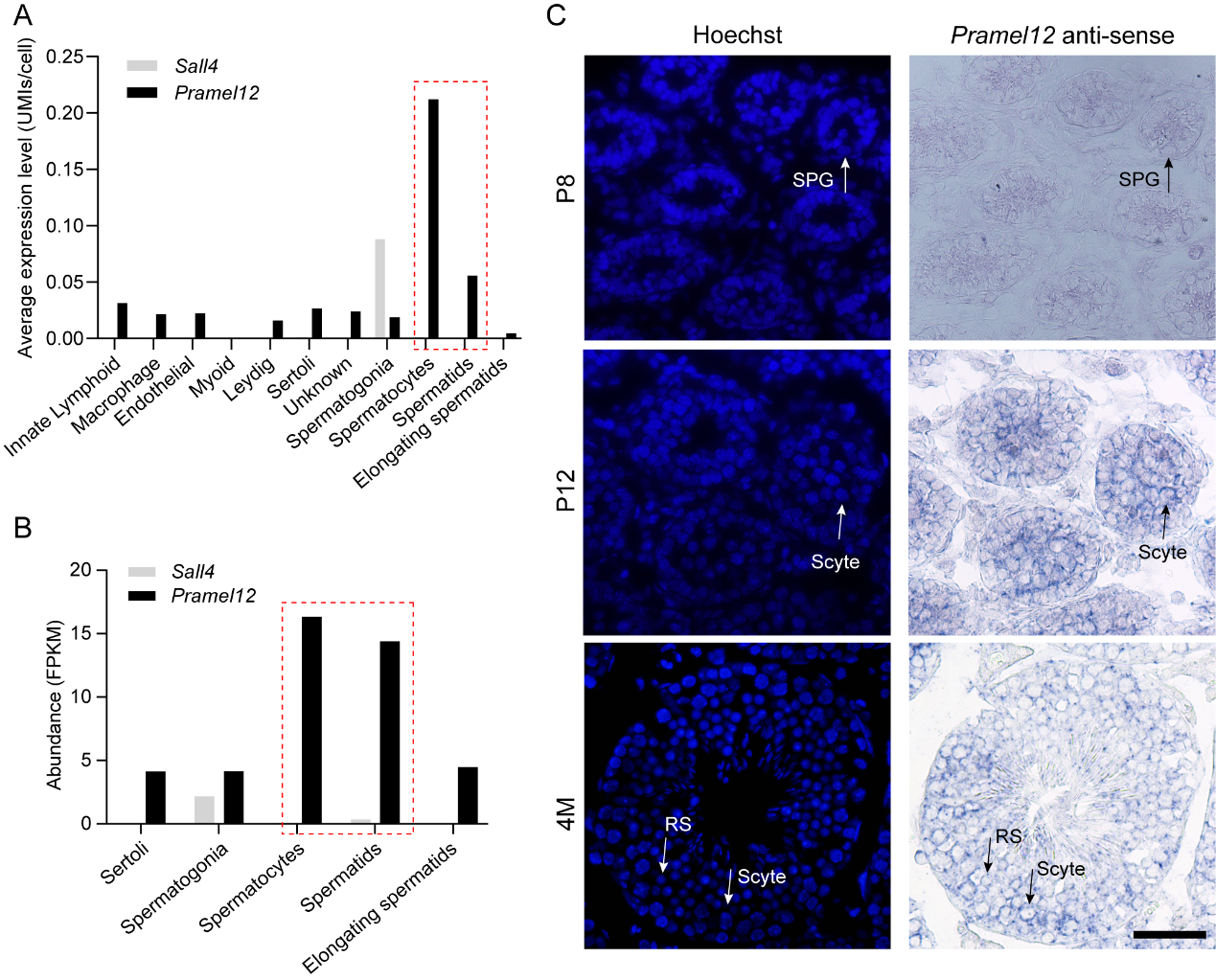
**

**Figure S1. *Pramel12* transcript expression in the mouse testes.** (A) The abundance of *Pramel12* transcripts in the eleven identified testicular cell types in adult mouse testes based on previously reported scRNA-seq data. Numbers on the Y axis reflect the average UMIs detected in individual cells. (B) Abundance of *Pramel12* transcripts in testicular cells, including Sertoli cells, spermatogonia, spermatocytes, spermatids, and elongating spermatids based on previously reported RNA-seq data. (C) *In situ* hybridization mRNA analysis of *Pramel12* in P8, P12, and 4-month-old wild-type mouse testes. DNA was stained with Hoechst 33342. SPG, Spermatogonia; Scyte, Spermatocyte; RS, round spermatid. Scale bar, 50 μm. Representative of n = 3 biologically independent replicates with similar results per condition.


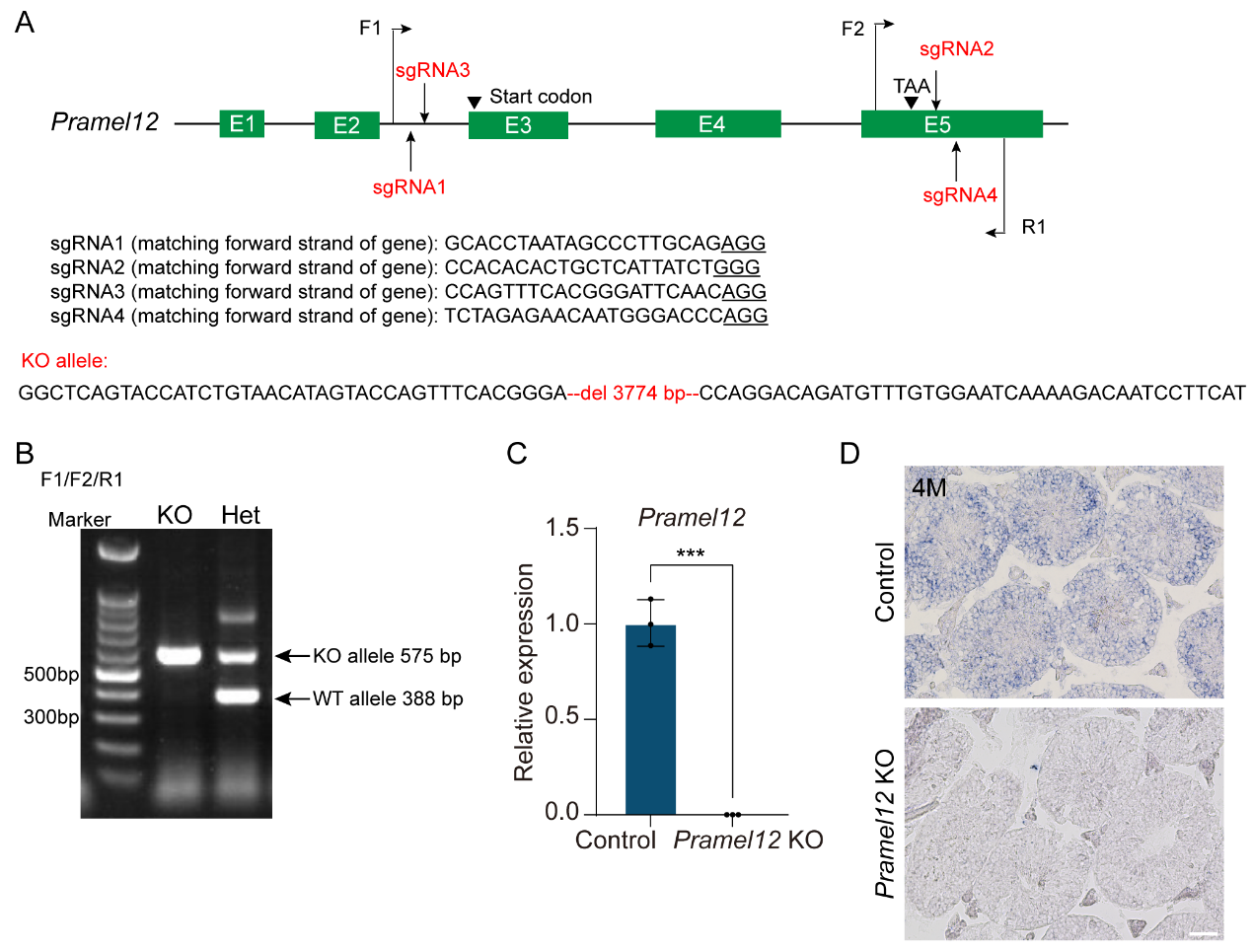


**Figure S2. Generation of *Pramel12*-null mice.** (A) Exon map of the mouse *Pramel12* locus. The position and sequence of the guide RNAs are shown. F1, F2, and R1 indicate the positions of the genotyping primers. (B) PCR genotyping was used to detect the wild-type (WT) and KO alleles. (C) Quantitative RT-qPCR analysis of *Pramel12* transcripts in adult control and *Pramel12*-null testes. For comparison, the abundance (relative to *β-actin*) of *Pramel12* in control testes was set to 1. Data are presented as mean ± s.d. for n = 3 biologically independent samples. ****P* < 0.001. (D) *In situ* hybridization mRNA analysis of *Pramel12* in adult control and *Pramel12* mutant testes. Scale bar, 50 μm. Representative of n = 3 independent biological replicates with similar results per condition.


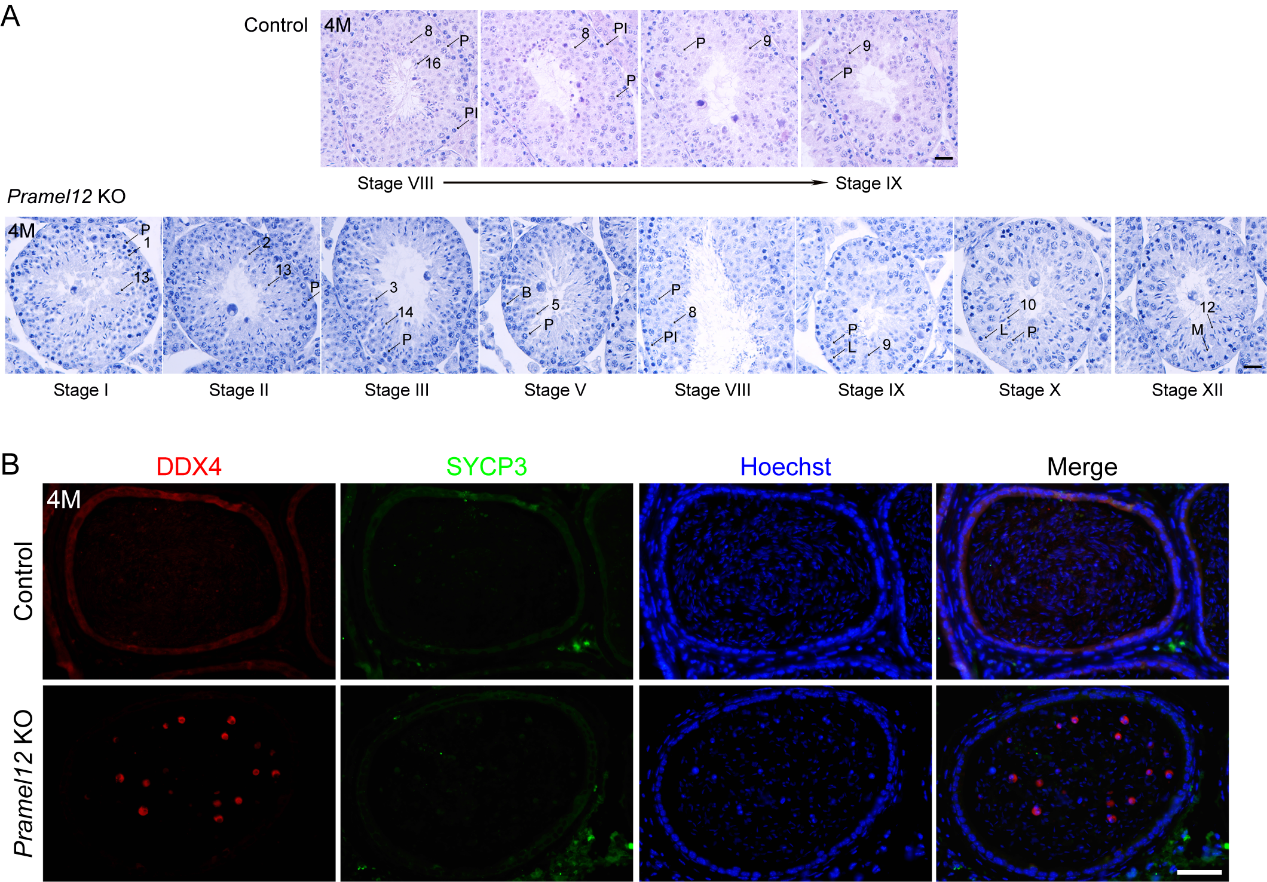


**Figure S3. Assessment of testes and cauda epididymides of 4-month-old *Pramel12*-null mice.** (A) Testicular sections of 4-month-old control and *Pramel12*-null mice were stained with hematoxylin-eosin. Tubules at different stages of the seminiferous cycle were indicated. The seminiferous epithelium of mice cycles through twelve stages. In *Pramel12* KO testes, a single cross-section reveals tubules at multiple stages, from which we selected tubules at stages V and XII (from the same section) for presentation to exemplify the phenotype. The numbers indicate spermatids at the corresponding developmental stages. B, Type B spermatogonia; Pl, Preleptotene; L, Leptotene; P, Pachytene; M, Metaphase. Scale bar, 50 μm. (B) Co-immunostaining of DDX4 and SYCP3 in adult control and *Pramel12*-null cauda epididymides. The DNA was stained with Hoechst 33342. Scale bar, 50 μm. Representative of n = 3 (A, B) independent biological replicates with similar results per condition.


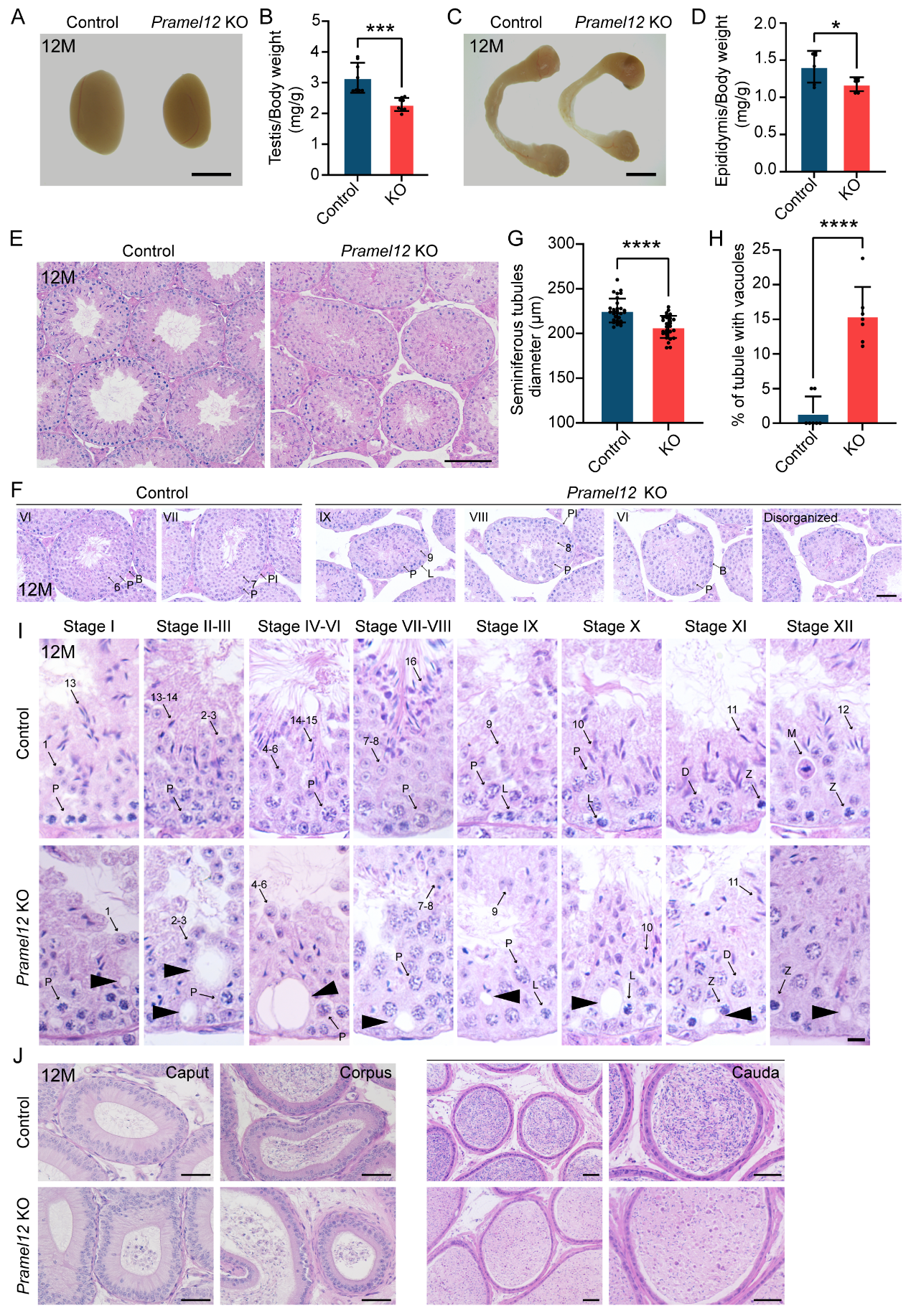


**Figure S4. PRAMEL12 is required for spermatogenesis.** (A, C) Morphology of testes (A) and epididymides (C) from 12-month-old control and *Pramel12*-null mice. Scale bars, 2 mm. (B, D) Testis-to-body weight ratio (B) and epididymis-to-body weight ratio (D) in 12-month-old control and *Pramel12* KO mice. Data represent mean ± s.d.; **P* < 0.05; ****P* < 0.001. (E, F) Histology of 12-month-old control and *Pramel12*-null testes. Scale bars: 100 μm (E), 50 μm (F). (G, H) Seminiferous tubule diameter (G) and percentage of vacuolated tubules (H) in 12-month-old control and *Pramel12*-null testes. Data represent mean ± s.d.; **** *P* < 0.0001. (I) H&E-stained seminiferous tubules at all twelve stages of the epithelial cycle in 12-month-old control and *Pramel12*-null mice. Arrowheads indicate vacuoles. Scale bar, 10 μm. (J) H&E-stained cross-sections of caput, corpus, and cauda epididymides from 12-month-old control and *Pramel12*-null mice. Scale bar, 50 μm. (F, I) The numbers indicate spermatids at the corresponding developmental stages. B, Type B spermatogonia; Pl, Preleptotene; L, Leptotene; Z, Zygotene; P, Pachytene; D, Diplotene; M, Metaphase.


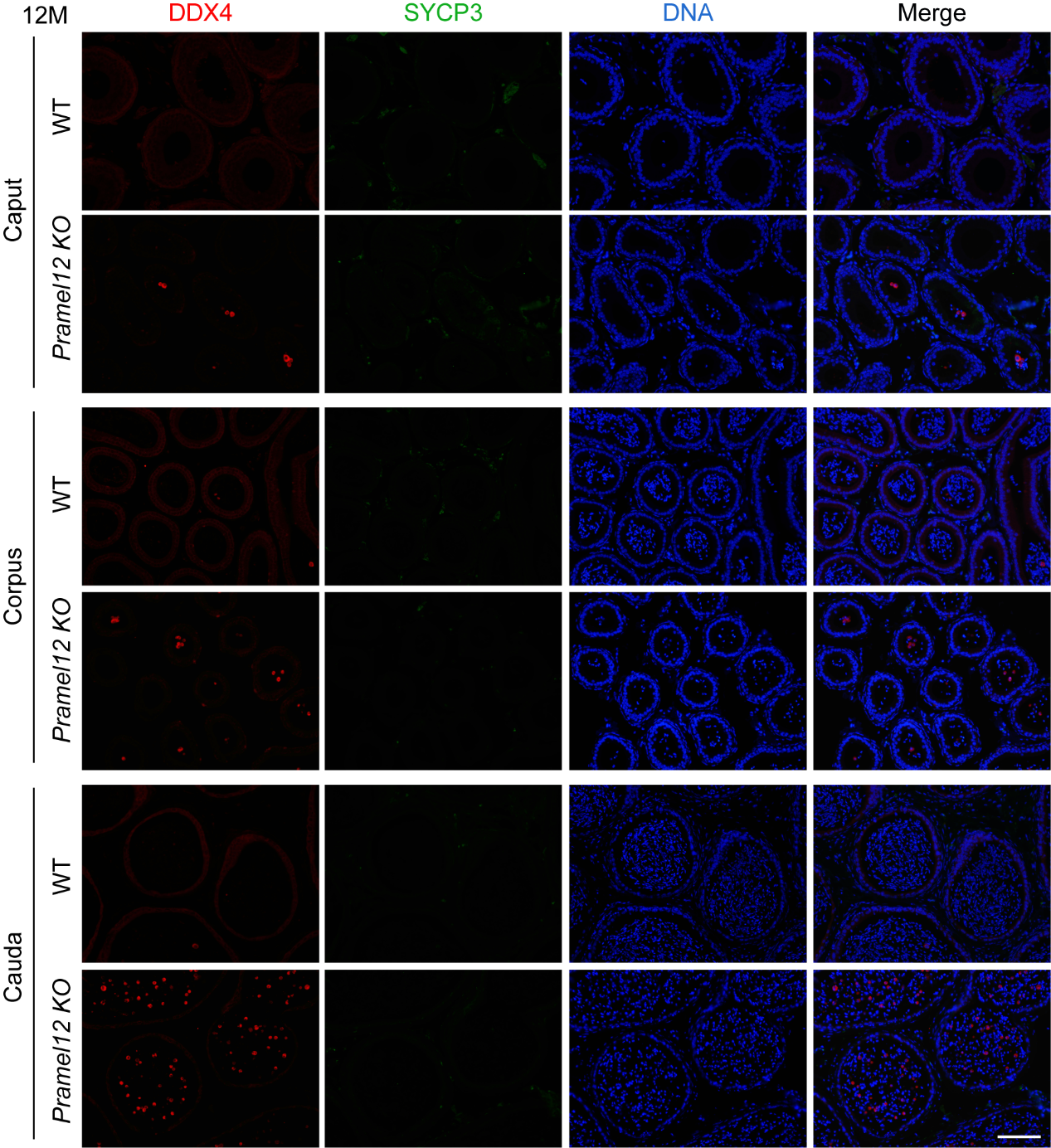


**Figure S5. Assessment of epididymides in 12-month-old *Pramel12*-null mice.** Co-immunostaining for DDX4 and SYCP3 in caput, corpus, and cauda epididymides from 12-month-old control and *Pramel12*-null mice. Nuclei were counterstained with Hoechst 33342. Scale bar, 100 μm.


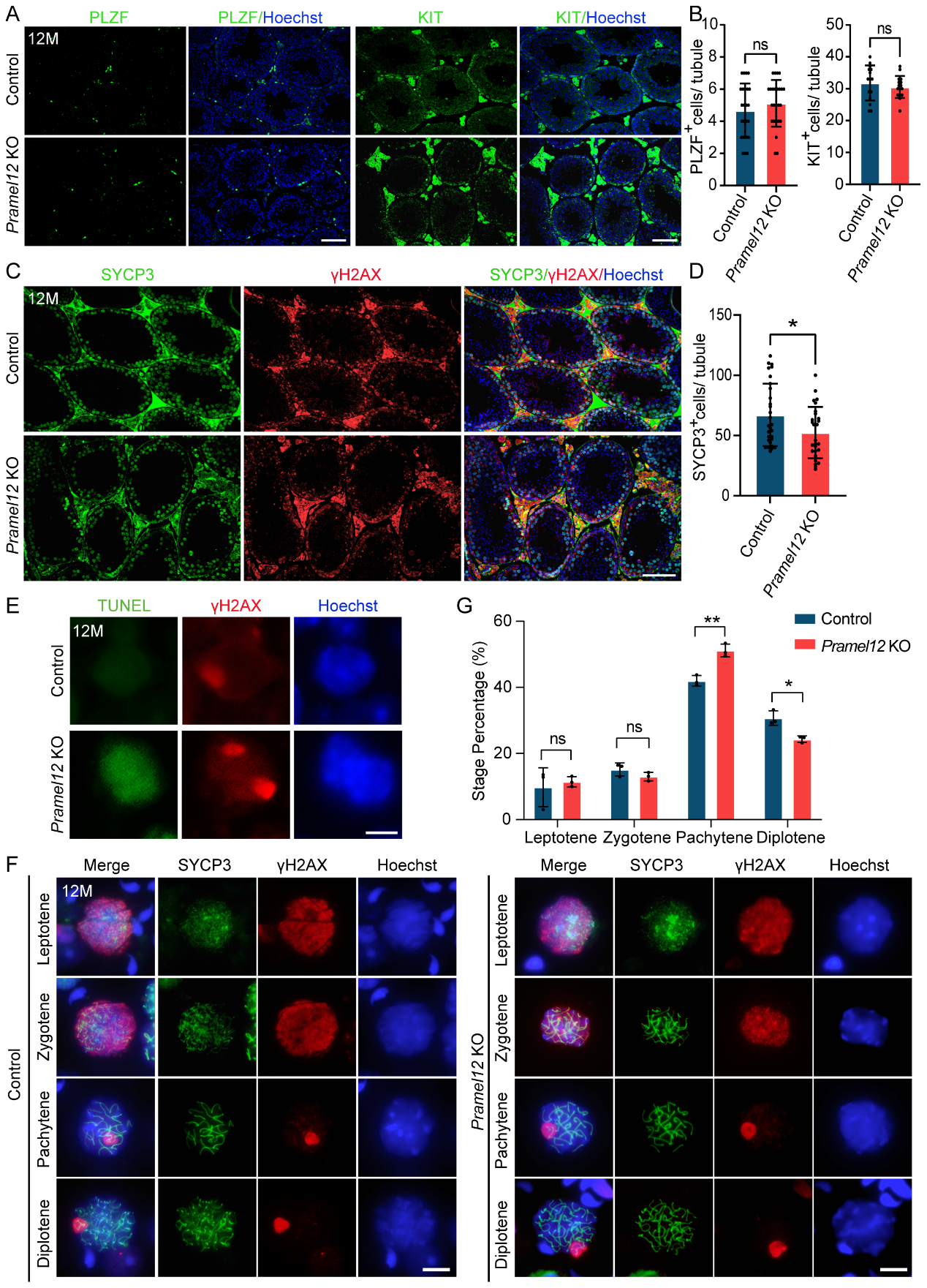


**Figure S6. PRAMEL12 is required for spermatocyte development and meiosis.** (A) Immunofluorescence staining for PLZF or KIT in 12-month-old control and *Pramel12*-null testes. DNA was counterstained with Hoechst 33342. Scale bar, 100 μm. (B) Quantification of PLZF-positive and KIT-positive cells per seminiferous tubule. (C) Co-immunofluorescence staining for SYCP3 and γH2AX in 12-month-old control and *Pramel12*-null testes. DNA was counterstained with Hoechst 33342. Scale bar, 100 μm. (D) Quantification of SYCP3-positive cells per seminiferous tubule. (E) Co-immunostaining for TUNEL and γH2AX in spermatocytes from 12-month-old control and *Pramel12*-null testes. DNA was counterstained with Hoechst 33342. Scale bar, 5 μm. (F) Chromosome spreads of spermatocytes immunostained for γH2AX and SYCP3. Scale bar, 10 μm. (G) Stage distribution quantification of spermatocytes (leptotene, zygotene, pachytene, diplotene). Data represent mean ± s.d. (panels B, D, G); ns, not significant; **P* < 0.05; ***P* < 0.01.


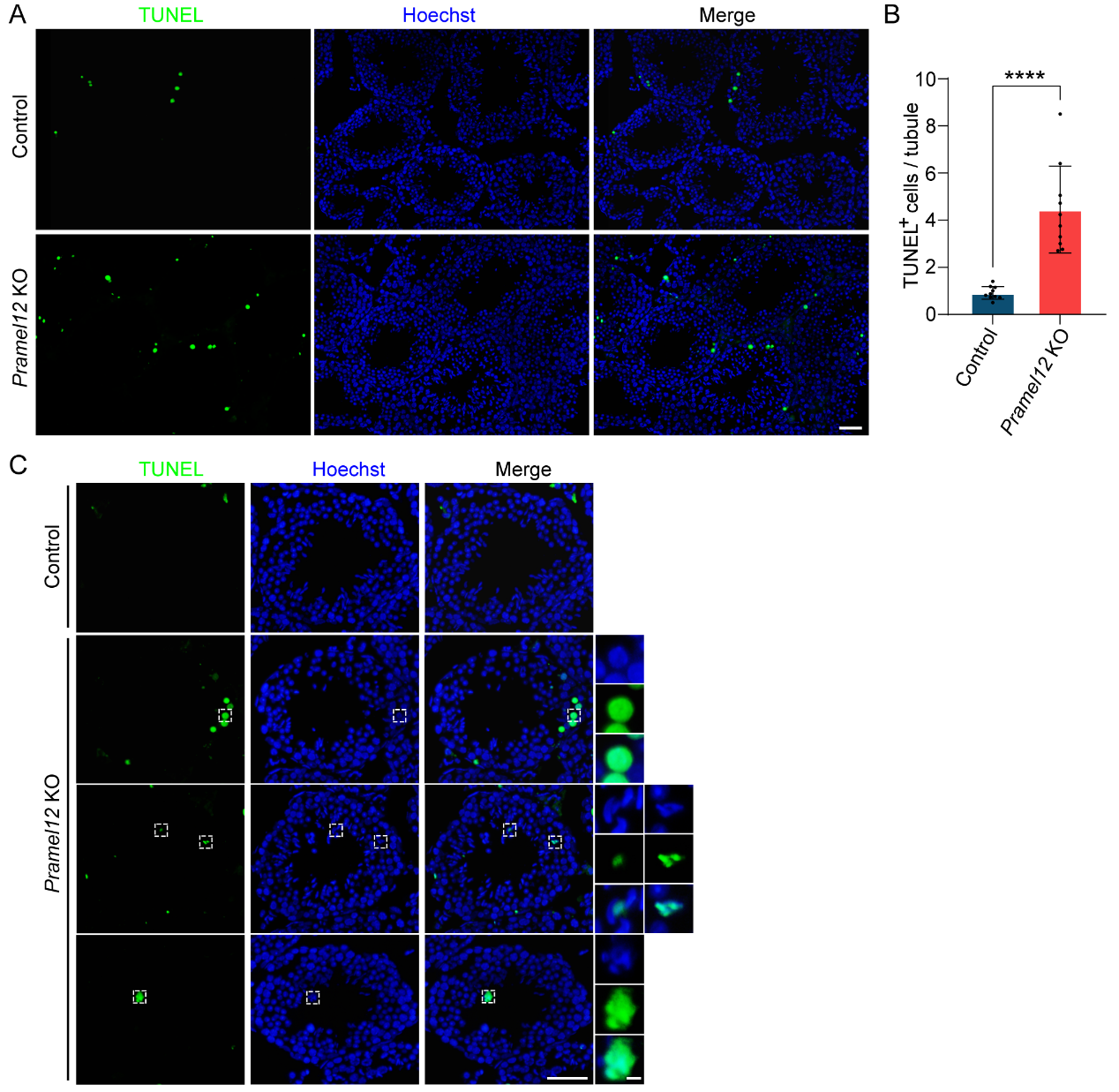


**Figure S7. Cell apoptosis in 12-month-old *Pramel12*-null testes.** (A) TUNEL assay on testicular sections from control and *Pramel12*-null mice. Nuclei counterstained with Hoechst 33342. Scale bar, 50 μm. (B) Quantification of TUNEL-positive cells per seminiferous tubule. Data represent mean ± s.d.; *****P* < 0.0001. (C) TUNEL analysis showing TUNEL-positive germ cells, including elongated spermatids, in *Pramel12*-null testes. Nuclei counterstained with Hoechst 33342. Scale bars: 50 μm (overview), 10 μm (insets).


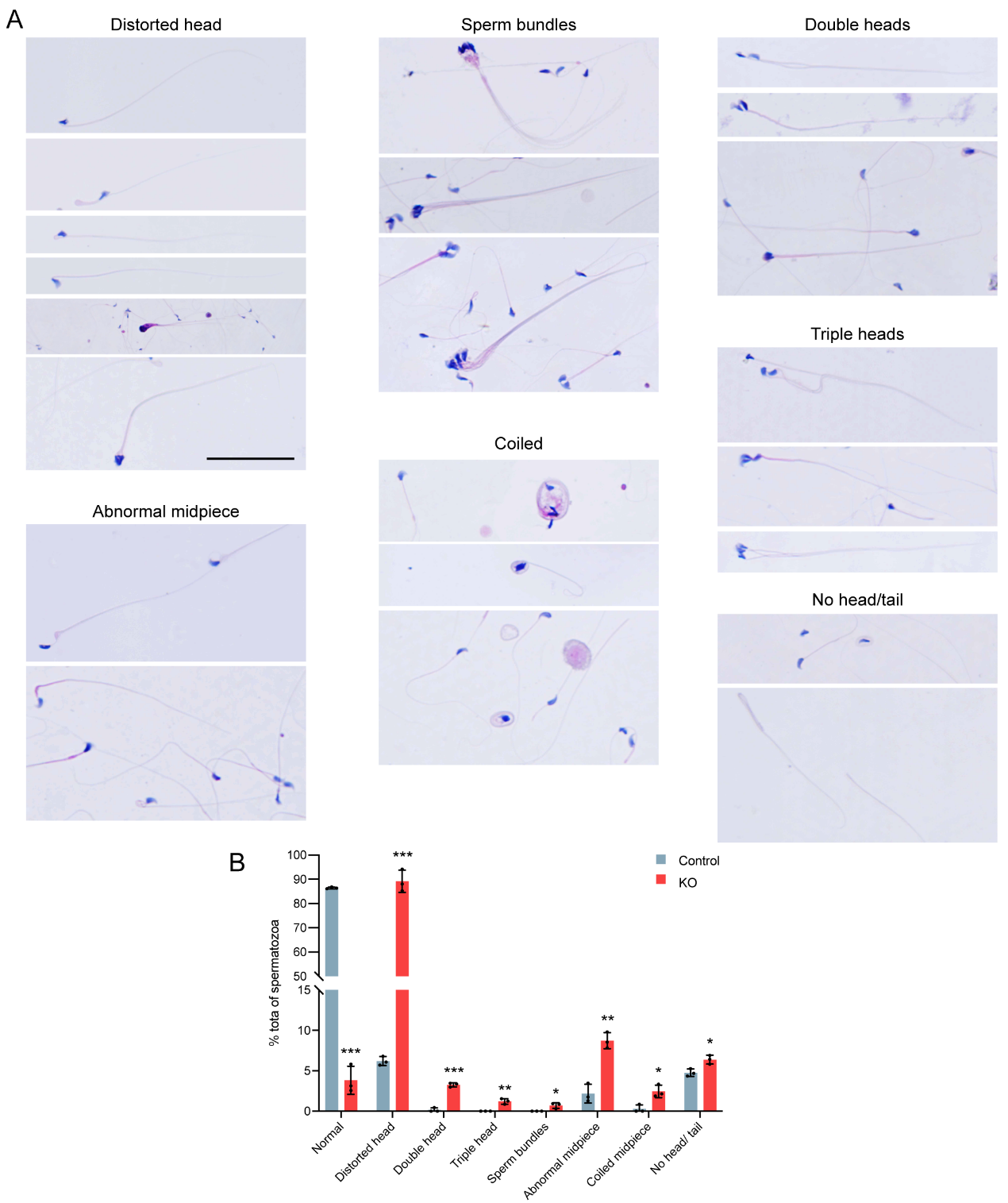


**Figure S8. Sperm morphology of adult *Pramel12*-null mice.** (A) Morphological analysis of sperm from 4-month-old *Pramel12*-null cauda epididymides. Scale bar, 50 μm. (B) Statistical analysis of the ratio of normal and malformed sperm in adult control and *Pramel12*-null mice. Data are presented as mean ± s.d.; **P* < 0.05; ***P* < 0.01; and ****P* < 0.001.


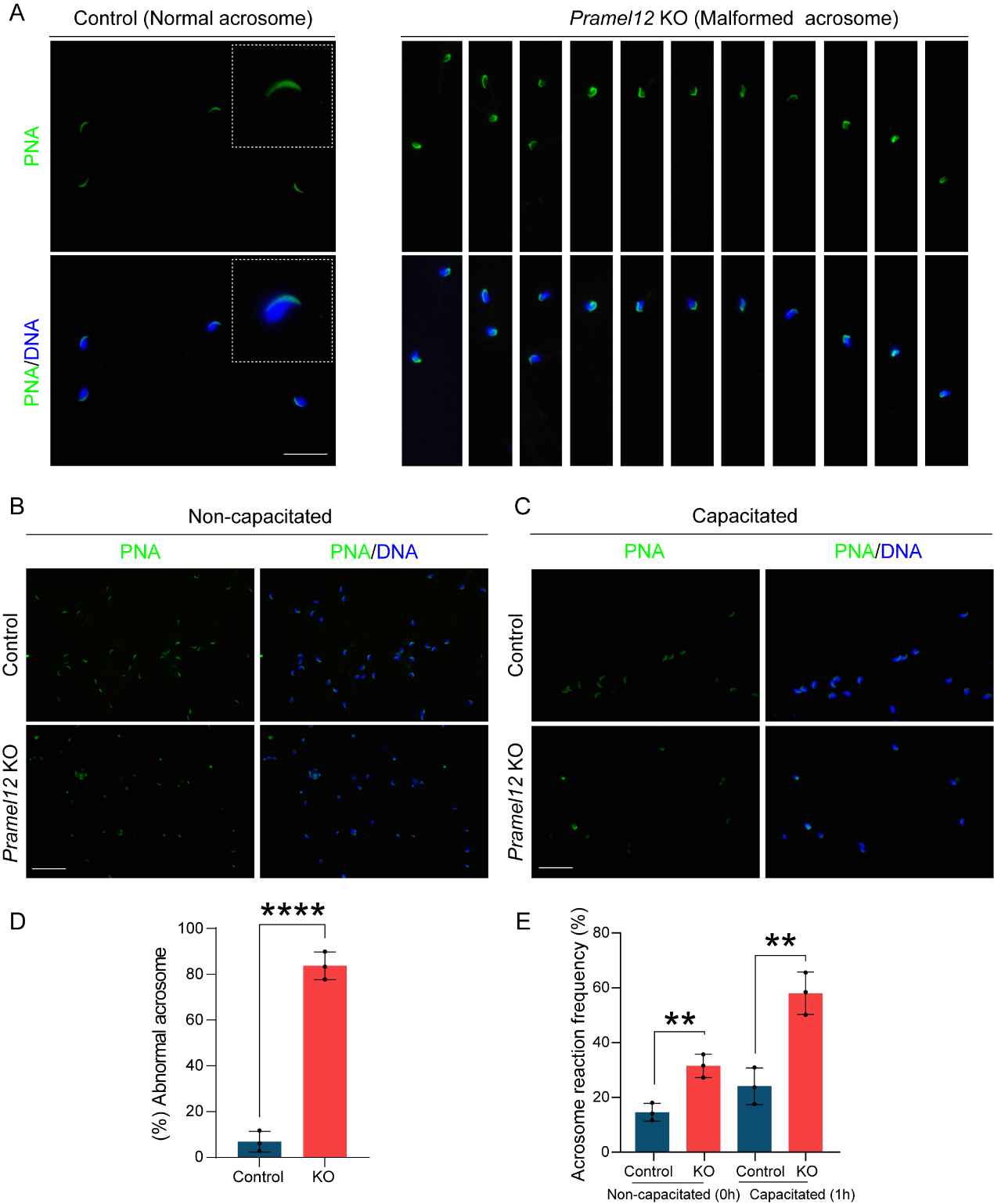


**Figure S9. Sperm acrosome and acrosome reaction frequency in *Pramel12*-null mice.** (A) Epididymal sperm from 4-month-old control and *Pramel12*-null mice were stained with PNA and Hoechst 33342. Scale bar, 20 μm. (B, C) Representative images of non-capacitated (0 h) or capacitated (1 h) sperm acrosome reaction in control and *Pramel12*-null mice, with the acrosome stained with PNA and the DNA stained with Hoechst 33342. Scale bar, 50 μm. (D) Statistical analysis of the ratio of abnormal acrosomes in control and *Pramel12*-null sperm. Data are presented as mean ± s.d.; *****P* < 0.0001. (E) Statistical analysis of non-capacitated (0 h) and capacitated (1 h) sperm acrosome reaction frequency in control and *Pramel12*-null mice. Data are presented as mean ± s.d.; ***P* < 0.01.


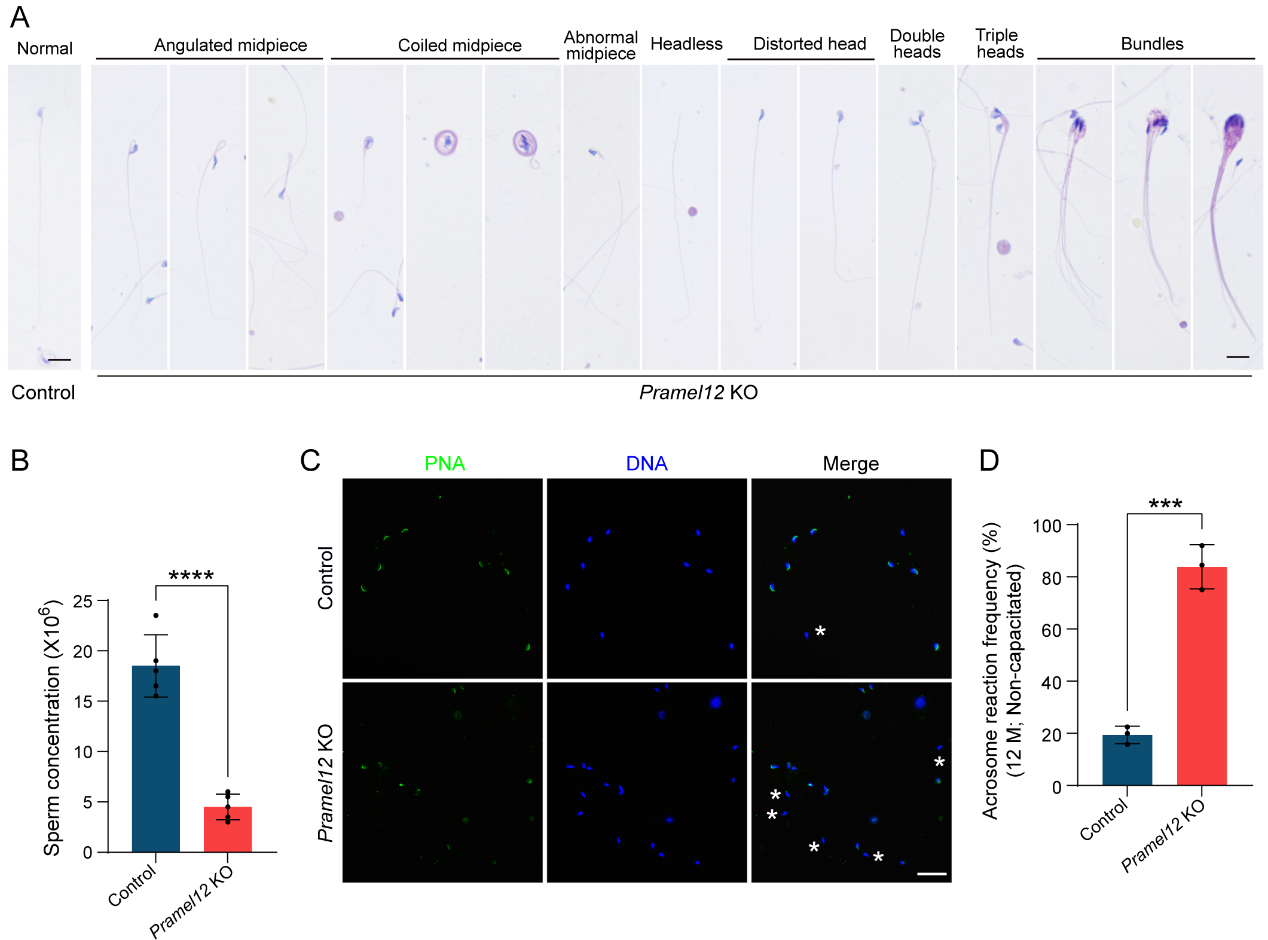


**Figure S10. Sperm morphology, concentration, and acrosome reaction in 12-month-old *Pramel12*-null males.** (A) Representative images of sperm morphology from 12-month-old control and *Pramel12*-null mice. Scale bar, 10 μm. (B) Epididymal sperm concentration. Data represent mean ± s.d.; *****P* < 0.0001. (C) Acrosome reaction frequency in non-capacitated sperm. Arrowheads indicate acrosome-reacted (PNA-negative) sperm. Scale bar, 50 μm. (D) Quantification of acrosome reaction frequency under non-capacitated condition. Data represent mean ± s.d.; ****P* < 0.001.


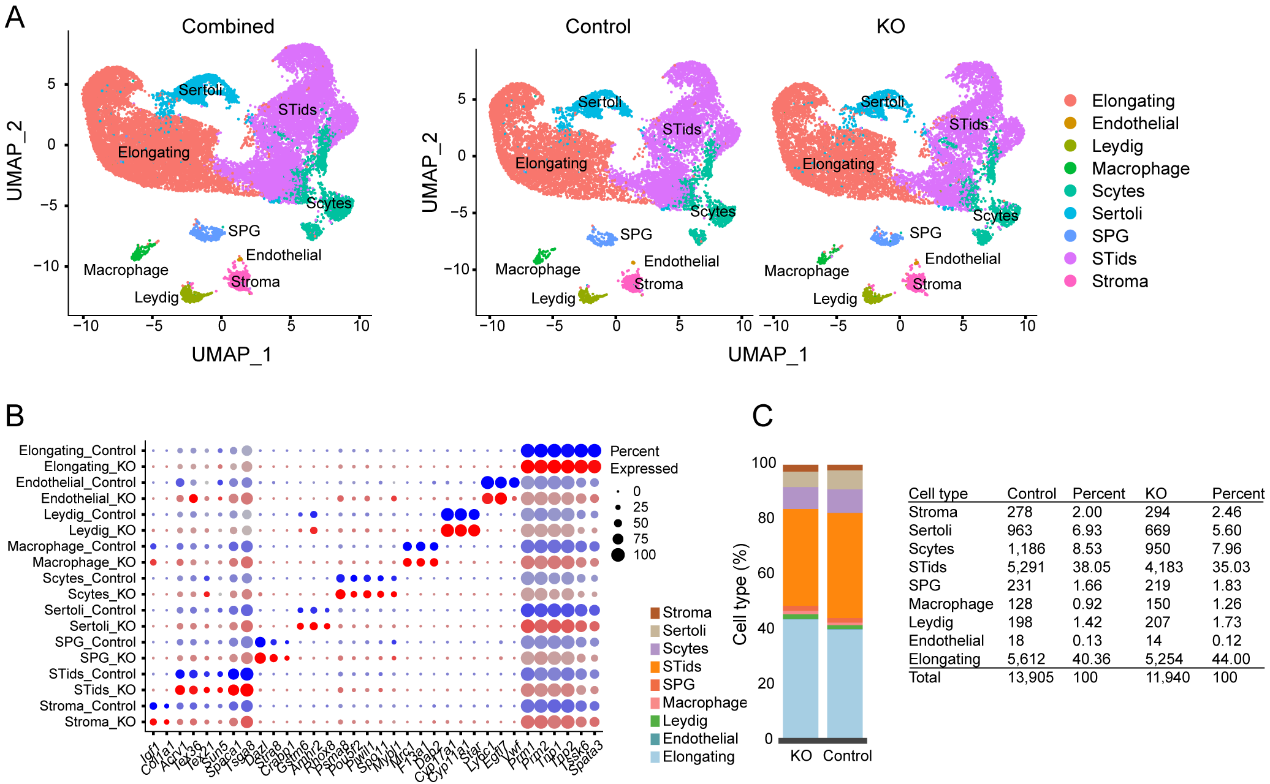


**Figure S11. scRNA-seq analysis of adult control and *Pramel12*-null testes.** (A) UMAP plots of nine identified cell types from combined (left panel), control (middle panel), and *Pramel12* KO (right panel) testicular cells. Each dot represents a single cell, and cell types are indicated by colors. (B) Dot plot for the selected marker genes corresponding to each cell type. (C) The number and percentage of each cell type in control and *Pramel12*-null testes.


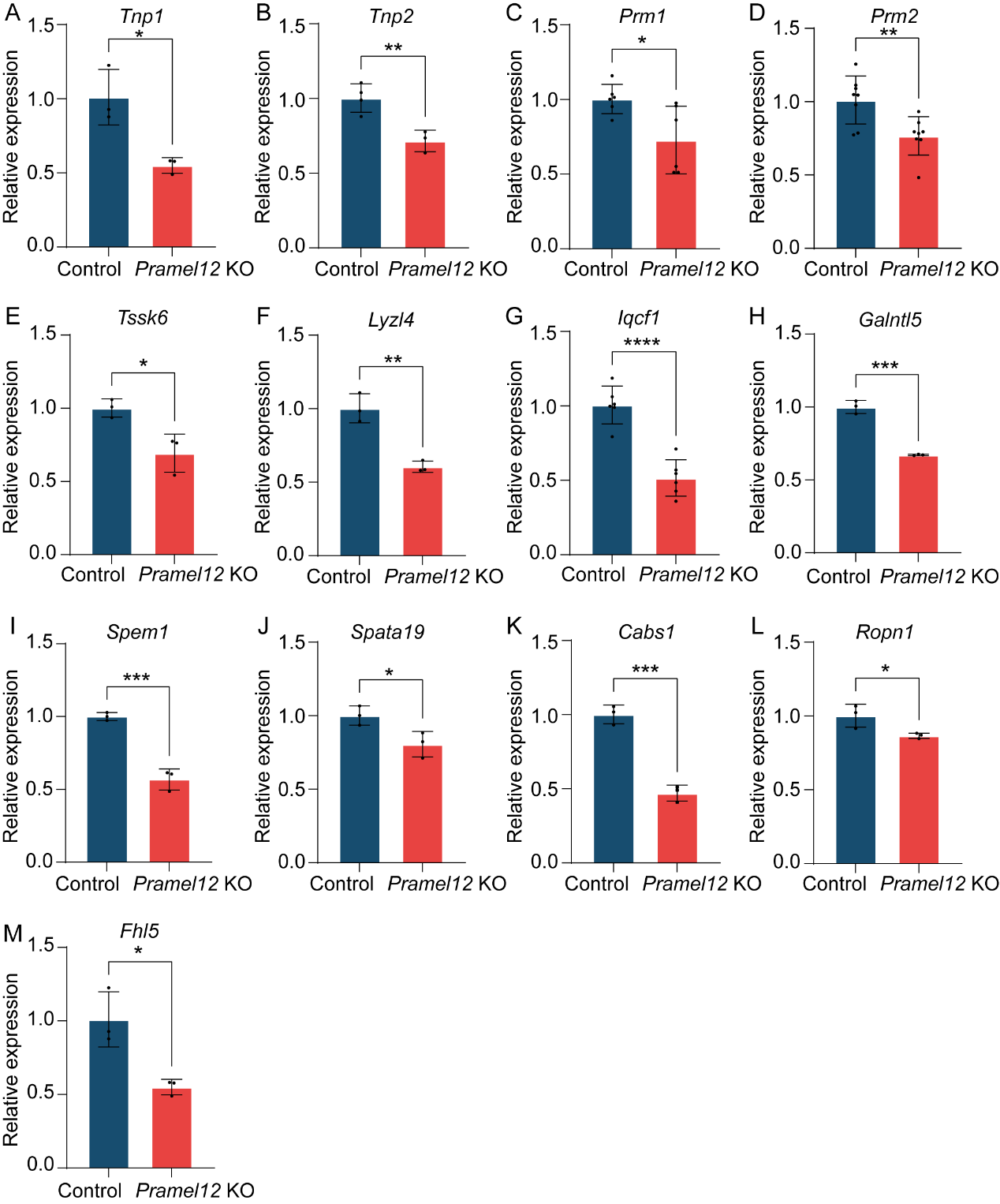


**Figure S12. The effect of PRAMEL12 deficiency on spermiogenic genes.** RT-qPCR analysis of (A) *Tnp1*, (B) *Tnp2*, (C) *Prm1*, (D) *Prm2*, (E) *Tssk6*, (F) *Lyzl4*, (G) *Iqcf1*, (H) *Galntl5*, (I) *Spem1*, (J) *Spata19*, (K) *Cabs1*, (L) *Ropn1*, and (M) *Fhl5* in 4-month-old control and *Pramel12*-null testes. The expression levels of genes in control relative to *β-actin* were set to 1. Data are presented as mean ± s.d.; **P* < 0.05; ***P* < 0.01; ****P* < 0.001; and *****P* < 0.0001.


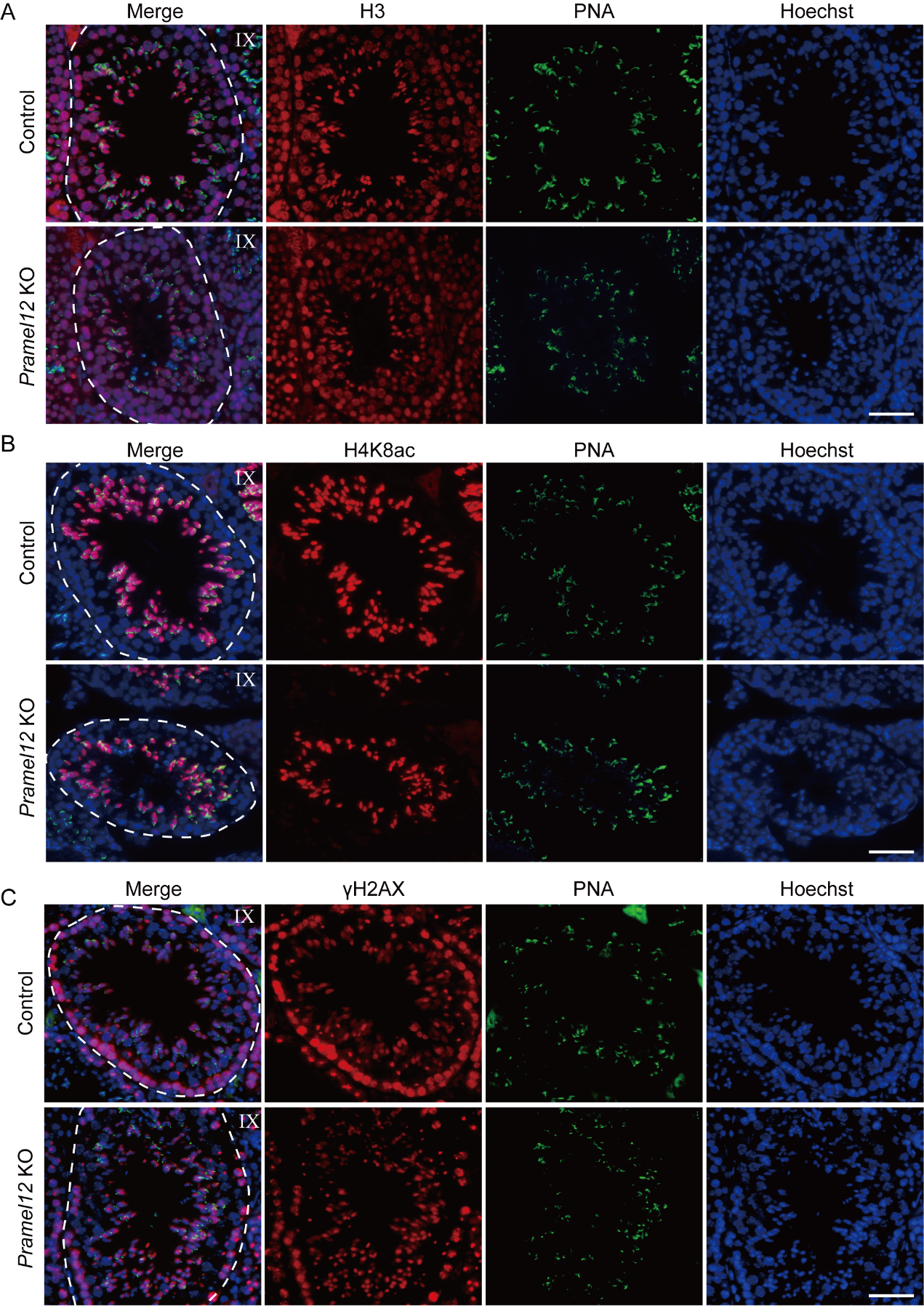


**Figure S13. Immunofluorescence analyses of H3, H4K8ac, and γH2AX in stage IX tubules.** (A) Co-immunostaining of H3 and PNA in stage IX seminiferous tubules of 4-month-old control and *Pramel12*-null mice. DNA was stained with Hoechst 33342. Scale bar, 50 μm. (B) Same as (A), but stained with antibodies against H4K8ac and PNA. (C) Same as (A), but stained with antibodies against γH2AX and PNA. Representative of n = 3 (A-C) independent biological replicates with similar results per condition.


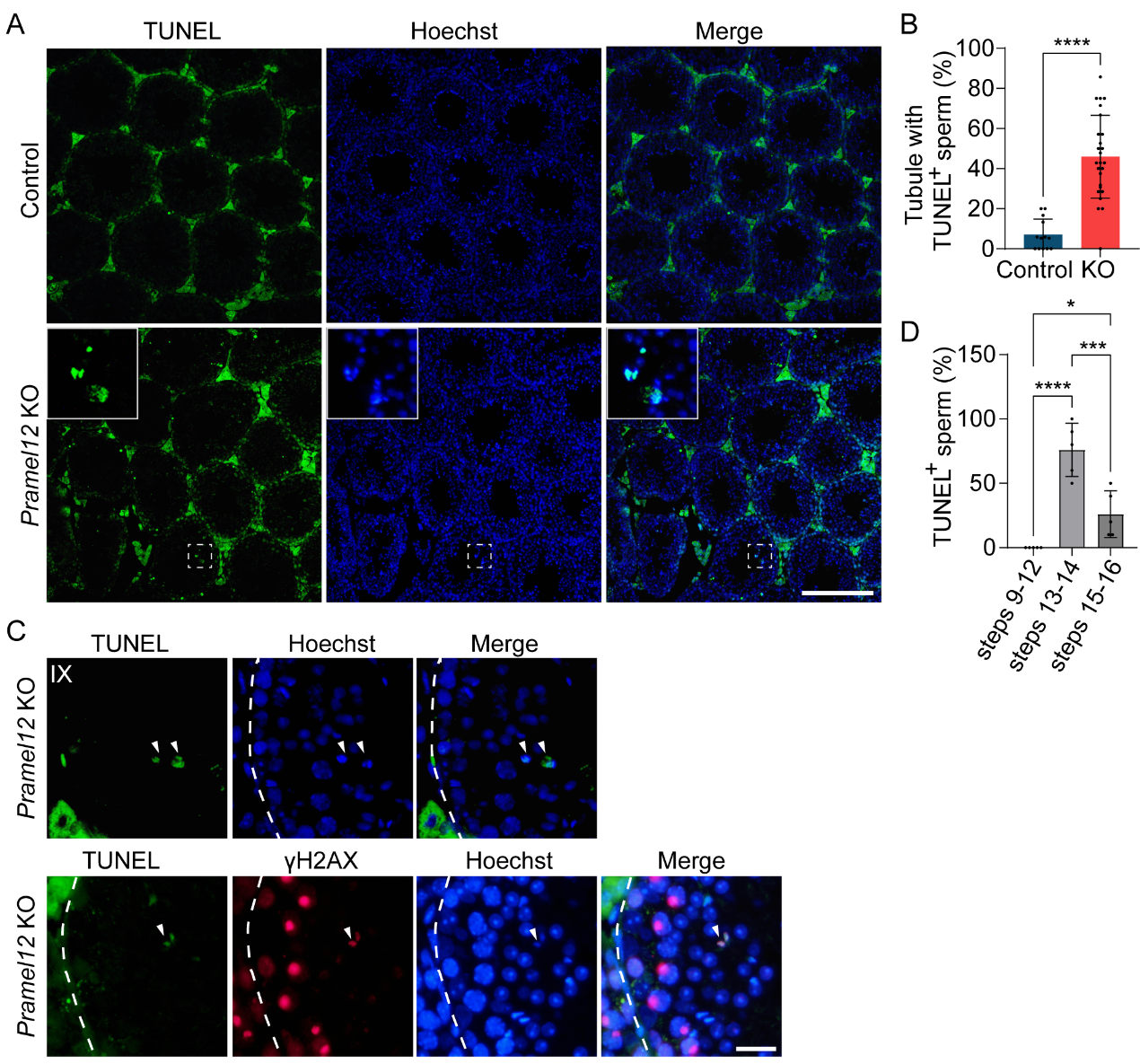


**Figure S14. TUNEL analysis of adult control and *Pramel12*-null testes.** (A) TUNEL assay on testicular sections of 4-month-old control and *Pramel12*-null testes. DNA was stained with Hoechst 33342. Scale bar, 100 μm. (B) Statistical analysis of the proportion of seminiferous tubules containing TUNEL-positive spermatids in control and *Pramel12*-null testes. (C) TUNEL assay on *Pramel12*-null testicular sections. Some unreleased spermatids in stage IX tubules were positive for TUNEL signals (upper panels). Some sperm were positive for both γH2AX and TUNEL signals (lower panels). DNA was stained with Hoechst 33342. Arrowheads indicate TUNEL-positive spermatids. Scale bar, 20 μm. (D) Bar graph showing the percentage of TUNEL-positive spermatids at indicated developmental steps. Data are presented as mean ± s.d. (B, D); **P* < 0.05; ****P* < 0.001; and *****P* < 0.0001.


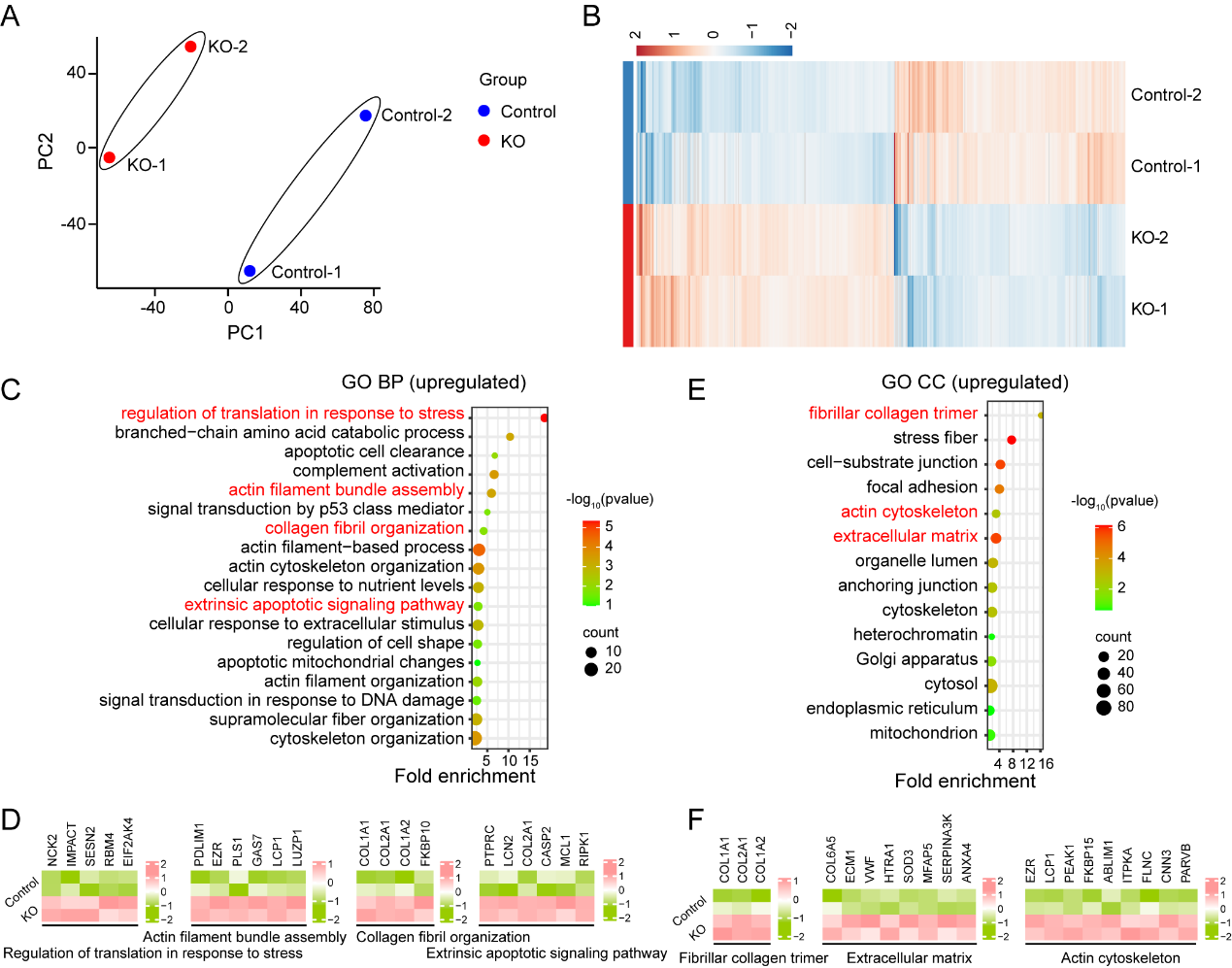


**Figure S15. Proteomic profiling of adult *Pramel12*-null testes.** (A) Principal component analysis (PCA) of testicular proteomes from 4-month-old control and *Pramel12* KO mice. (B) Heatmap of differentially expressed proteins between control and *Pramel12*-null testes. (C) Enriched biological process GO terms for upregulated proteins. (D) Heatmaps of differentially expressed proteins corresponding to selected panel (C) GO terms. (E) Enriched cellular component GO terms for upregulated proteins. (F) Heatmaps of differentially expressed proteins corresponding to selected panel (E) GO terms.


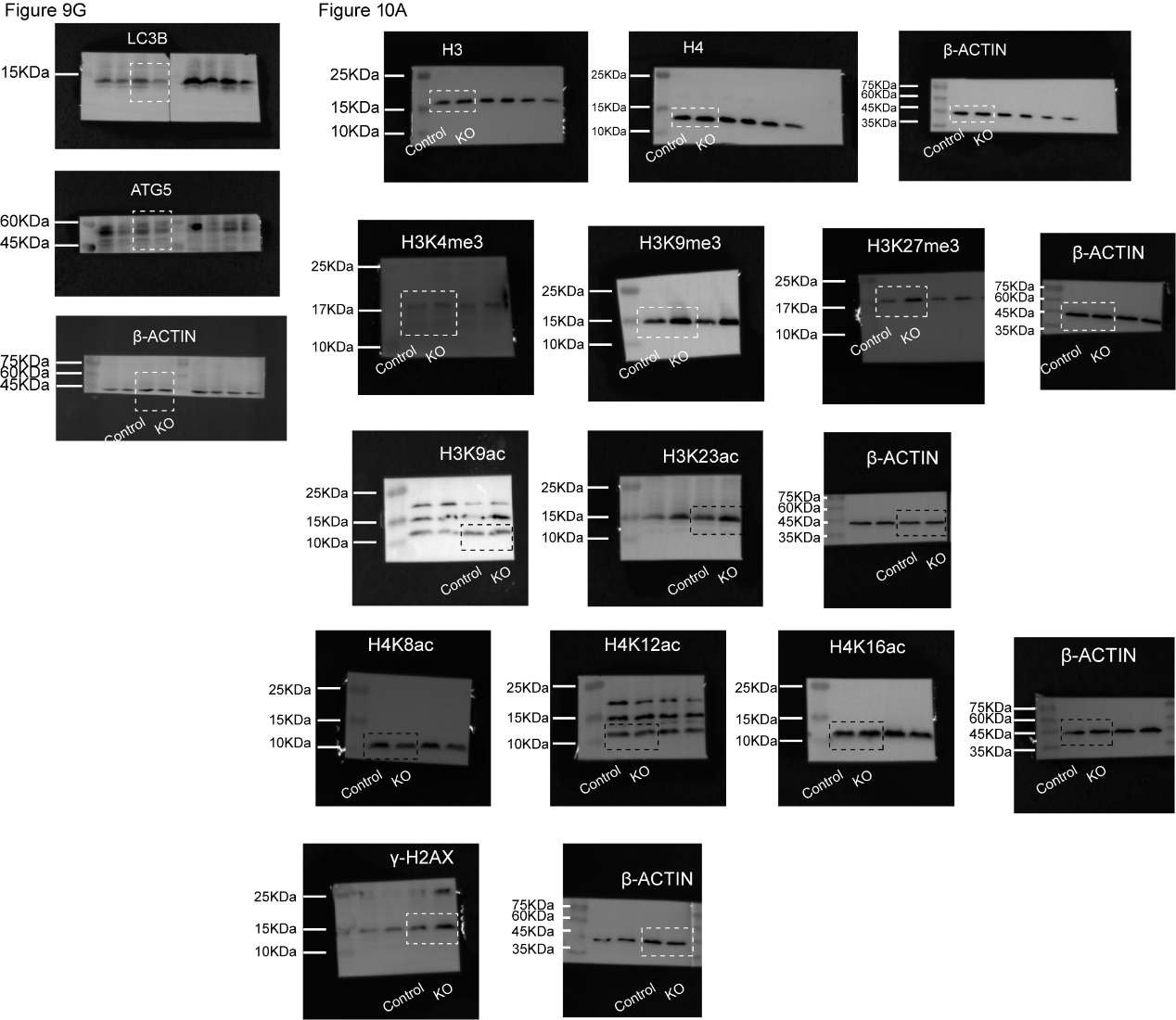


**Figure S16. Uncropped immunoblot images in this study.** Uncropped immunoblot images for Figure 9G and Figure 10A.


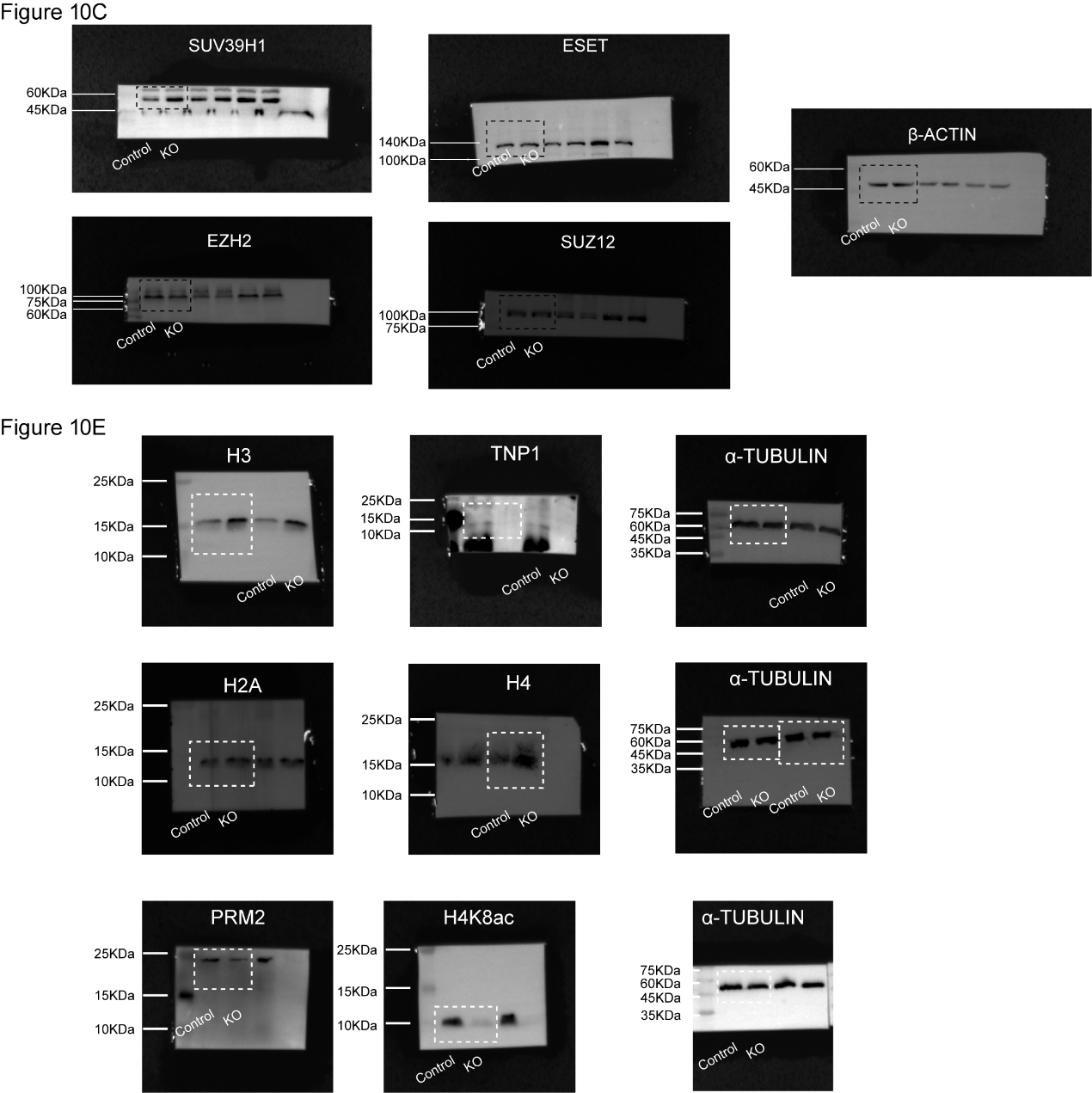


**Figure S17. Uncropped immunoblot images in this study.** Uncropped immunoblot images for Figure 10C and E.

**Table S1. Genotyping primers for *Pramel12* KO mice**

| Gene | Direction | Primer (5’-3’) |
| --- | --- | --- |
| WT band 644 bp | F^1^ | TGTCCAACTTTGTCCTGAGCTTAT |
|  | R | ACCATGTGAGAAGGAGAAAGTCAT |
| KO band 575 bp | F | TGAGAATCCCACATCAAGCTCTAAA |
|  | R | ACCATGTGAGAAGGAGAAAGTCAT |

^1^F, forward; R, reverse

**Table S2. Antibodies used in this study**

| Antibody | Company | Identifier | Immunohisto-chemistry | Immunoblot |
| --- | --- | --- | --- | --- |
| Rabbit anti-DDX4 | Abcam | Cat# ab13840 | 1:200 |  |
| Mouse anti-SYCP3 | Abcam | Cat# ab97672 | 1:200 |  |
| Goat anti-PLZF | R&D Systems | Cat# AF2944 | 1:200 |  |
| Goat anti-KIT | R&D Systems | Cat# AF1356 | 1:200 |  |
| Rabbit anti-H2A | Selleck | Cat# F0687 |  | 1:1000 |
| Mouse anti-H3 | Beyotime | Cat# AF0009 |  | 1:1000 |
| Rabbit anti-H4 | Affinity | Cat# AF0009 |  | 1:1000 |
| Rabbit anti-TNP1 | Proteintech | Cat# 17178-1-AP |  | 1:1000 |
| Rabbit anti-PRM2 | Proteintech | Cat# 14500-1-AP |  | 1:1000 |
| Rabbit anti-H4K8ac | Beyotime | Cat# AF5626 |  | 1:1000 |
| Rabbit anti-H4K12ac | Affinity | Cat# AF1021 |  | 1:1000 |
| Rabbit anti-H4K16ac | Affinity | Cat# AF3636 |  | 1:1000 |
| Rabbit anti-H3K9ac | Affinity | Cat# AF3359 |  | 1:1000 |
| Rabbit anti-H3K23ac | Affinity | Cat# AF1019 |  | 1:1000 |
| Rabbit anti- H3K4me3 | Abmart | Cat# P37961-7F |  | 1:1000 |
| Rabbit anti- H3K9me3 | ABclonal | Cat# A2360 |  | 1:500 |
| Rabbit anti- H3K27me3 | Abways | Cat# CY9075 |  | 1:1000 |
| Rabbit anti-SUZ12 | Abways | Cat# CY5990 |  | 1:1000 |
| Rabbit anti-EZH2 | Selleck | Cat# F0281 |  | 1:1000 |
| Rabbit anti-ESET | Selleck | Cat# F1329 |  | 1:1000 |
| Rabbit anti-SUV39H1 | Cell Signaling Technology | Cat# 8729T |  | 1:1000 |
| Rabbit anti-LC3B | Abmart | Cat# T55992F |  | 1:1000 |
| Rabbit anti-ATG5 | Abmart | Cat# T55766F |  | 1:1000 |
| Mouse anti-α-TUBULIN | ABclonal | Cat# AC012 | 1:500 | 1:5000 |
| Rabbit anti-ACTIN | ABclonal | Cat# AC026 |  | 1:100000 |
| Rabbit anti-phospho-histone H2A.X (Ser139) | Cell Signaling Technology | Cat# 9718 | 1:200 |  |
| Donkey anti-mouse IgG, Alexa Fluor 488 | Thermo Fisher Scientific | Cat# A-21202 | 1:200 |  |
| Donkey anti-rabbit IgG, Alexa Fluor 594 | Thermo Fisher Scientific | Cat# A-21207 | 1:200 |  |
| Donkey anti-goat IgG, Alexa Fluor 488 | Thermo Fisher Scientific | Cat# A-11055 | 1:200 |  |
| Goat anti-rabbit IgG, HRP | Thermo Fisher Scientific | Cat# 31460 |  | 1:5000 |
| Goat anti-mouse IgG, HRP | Abcam | Cat# 205719 |  | 1:5000 |

**Table S3. Primers used for RT-PCR**

| Gene | Direction | Primer (5’-3’) |
| --- | --- | --- |
| *Tnp1* | F^1^ | ACCAGCCGCAAGCTAAAGAC |
|  | R | TTTCCTACTTTTCAGGACGCTC |
| *Tnp2* | F | TCGACACTCACCTGCAAGAC |
|  | R | ATCCTGGAGTGCGTCACTTG |
| *Prm1* | F | ATGGCCAGATACCGATGCTG |
|  | R | GCAGCATCTTCGCCTCCTC |
| *Prm2* | F | GAGCGCGTAGAGGACTATGG |
|  | R | ATCTTCTGCAGCCTCTGCGAT |
| *Tssk6* | F | CGGGCGACAAACTCCTGAG |
|  | R | ACCGTCCCTTTATACTTCTTGGA |
| *Lyzl4* | F | GGGGCTTGAATTATTTTGAGGGC |
|  | R | GAGCCATCTTGTGGGTCCT |
| *Iqcf1* | F | ACTTAATGCACCTACTGACGATG |
|  | R | CCGACGTACCAGTGTACCG |
| *Galntl5* | F | CACCAGCAAACCCGTCACT |
|  | R | ATGGCATTTAATCCATATCGCCT |
| *Spem1* | F | CACCCACTCGTCGTCGTTAC |
|  | R | GCCTTCCCAATCCTTAGAGTCA |
| *Spata19* | F | ATCTTTGCCCGGAAAACCGTA |
|  | R | AGCTTCGCTTTCTTCAACTTCA |
| *Cabs1* | F | ATTCTCGACCTCCAAGAGACA |
|  | R | CCATCCGGTGTGCAGTCATTT |
| *Ropn1* | F | GAAGCAGTTTACCAAAGATGCCA |
|  | R | AGGGATTTGTTCAGACCTCTCT |
| *Fhl5* | F | TTGTGAGCAGTGTAAAGAACCAA |
|  | R | ACCAAAGAGTGATGGCATTTGTT |
| *Pramel12* | F | CAAGCATACTTGTTACAGTGGGC |
|  | R | CCAGGTAAGATGCAAAATGCAC |
| *β-actin* | F | GGCTGTATTCCCCTCCATCG |
|  | R | CCAGTTGGTAACAATGCCATGT |

^1^F, forward; R, reverse
